# Total Energy Expenditure, Energy Intake, and Body Composition in Endurance Athletes Across the Training Season: A Systematic Review

**DOI:** 10.1186/s40798-017-0076-1

**Published:** 2017-02-04

**Authors:** Juliane Heydenreich, Bengt Kayser, Yves Schutz, Katarina Melzer

**Affiliations:** 1 Swiss Federal Institute of Sport Magglingen SFISM, Hauptstrasse 247, 2532 Magglingen, Switzerland; 2 Faculty of Biology and Medicine, University of Lausanne, Lausanne, 1015 Switzerland; 3 Faculty of Medicine, University of Fribourg, Fribourg, 1700 Switzerland

## Abstract

**Background:**

Endurance athletes perform periodized training in order to prepare for main competitions and maximize performance. However, the coupling between alterations of total energy expenditure (TEE), energy intake, and body composition during different seasonal training phases is unclear. So far, no systematic review has assessed fluctuations in TEE, energy intake, and/or body composition in endurance athletes across the training season.

The purpose of this study was to (1) systematically analyze TEE, energy intake, and body composition in highly trained athletes of various endurance disciplines and of both sexes and (2) analyze fluctuations in these parameters across the training season.

**Methods:**

An electronic database search was conducted on the SPORTDiscus and MEDLINE (January 1990–31 January 2015) databases using a combination of relevant keywords.

Two independent reviewers identified potentially relevant studies. Where a consensus was not reached, a third reviewer was consulted. Original research articles that examined TEE, energy intake, and/or body composition in 18–40-year-old endurance athletes and reported the seasonal training phases of data assessment were included in the review. Articles were excluded if body composition was assessed by skinfold measurements, TEE was assessed by questionnaires, or data could not be split between the sexes.

Two reviewers assessed the quality of studies independently. Data on subject characteristics, TEE, energy intake, and/or body composition were extracted from the included studies. Subjects were categorized according to their sex and endurance discipline and each study allocated a weight within categories based on the number of subjects assessed. Extracted data were used to calculate weighted means and standard deviations for parameters of TEE, energy intake, and/or body composition.

**Results:**

From 3589 citations, 321 articles were identified as potentially relevant, with 82 meeting all of the inclusion criteria. TEE of endurance athletes was significantly higher during the competition phase than during the preparation phase (*p* < 0.001) and significantly higher than energy intake in both phases (*p* < 0.001). During the competition phase, both body mass and fat-free mass were significantly higher compared to other seasonal training phases (*p* < 0.05).

**Conclusions:**

Limitations of the present study included insufficient data being available for all seasonal training phases and thus low explanatory power of single parameters. Additionally, the classification of the different seasonal training phases has to be discussed.

Male and female endurance athletes show important training seasonal fluctuations in TEE, energy intake, and body composition. Therefore, dietary intake recommendations should take into consideration other factors including the actual training load, TEE, and body composition goals of the athlete.

**Electronic supplementary material:**

The online version of this article (doi:10.1186/s40798-017-0076-1) contains supplementary material, which is available to authorized users.

## Key Points

- Endurance athletes show training seasonal fluctuations in TEE, energy intake, and body composition.
- Dietary recommendations should consider the actual training load, TEE, and body composition goals.

## Background

Total energy expenditure (TEE) is composed of the energy costs of the processes essential for life (basal metabolic rate (BMR), 60–80% of TEE), of the energy expended in order to digest, absorb, and convert food (diet-induced thermogenesis, ~10%), and the energy expended during physical activities (activity energy expenditure, ~15–30%) [1, 2]. Elite endurance athletes are characterized by high fluctuations of TEE, mainly due to the variability of the energy expended during sporting activities. Among elite senior endurance athletes, training loads from 500 h/year [3, 4] up to 1000 h/year [5–7] have been reported, depending on the specific muscular loading characteristic of the sport. During heavy sustained exercise (e.g., during the Tour de France), TEE can be as high as fivefold the BMR over several weeks [8]. On the other hand, during recovery days, pre-competition tapers, or during the off-season, the energy expended in activities is far less. Therefore, TEE is expected to be much lower and may even reach levels comparable to that of sedentary behavior.

An appropriate energy intake supports optimal body function, determines the capacity for intake of macronutrients and micronutrients, and assists in manipulating body composition in athletes [9]. It is a challenge for each endurance athlete to appropriately match energy intake and TEE in order to achieve energy balance and thus, weight stability, both on a micro level (i.e., over 1 day or several days) and through the training and competitive season. Furthermore, endurance athletes in general strive for a low body mass and/or body fat level for various advantages in their sports, specifically during the competition season [10]. This allows runners and cyclists to reach greater economy of movement and better thermoregulatory capacity from a favorable ratio of weight to surface area and less insulation from subcutaneous fat tissue. Elite endurance athletes are therefore characterized by low body mass and body fat content. For example, in elite Kenyan endurance runners, the body fat percentage was 7.1% [11], which is only marginally above the recommended 5% minimum for males [12]. In the same athletes, body mass index (BMI) was 18.3 kg/m^2^ [11], which is generally classified as being underweight [13]. However, these athletes were in peak physical conditions as the investigations were undertaken and a low body fat percentage and body weight might be an advantage for competition. Achieving a negative energy balance and a concomitant loss of body and fat masses in preparation for competition can be accomplished in phases with high daily TEE solely by the reduction of energy intake, since any further training load increases could cause overtraining [12]. Therefore, the nutritional goals and requirements of endurance athletes are not static over the training year. Since endurance athletes undertake a periodized training program and follow periodized body composition goals, the nutritional support also needs to be periodized [9].

**Fig. 1:**
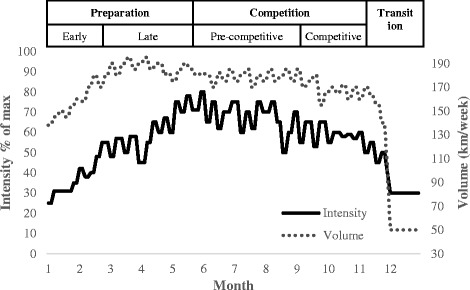
Periodization of the training year for a “one-peak annual year” of an elite runner. Adapted from Bompa & Haff [16]

Usually, the annual training schedule of an elite endurance athlete is divided into distinct phases, each with very specific objectives. This is necessary to maximize physiological adaptations for improved performance, usually scheduled to peak around the main competitions of the year [14]. The principle of training periodization was first introduced in the 1960s by the Soviet trainer Leo Matveyev [15] and has not fundamentally changed since then [14]. The basis of this model is to prepare the athlete for one or more major competitions during the year by separating the training into the following three main phases (macrocycles): preparatory, competitive, and transition phases [15]. An example for a “one-peak annual plan” for a runner is shown in Fig. 1. The preparatory phase is characterized by predominantly high-volume training at moderate intensities, which improves endurance capacity and provides a more efficient use of fuel substrates. During the late preparatory phase, training volume is reduced while intensity is gradually increased. The goal of this phase is to reach peak performance and to transfer the training effects into the competitive phase, where exercise intensity is the highest. In the week before an important competition, volume and intensity are typically decreased (taper phase) to allow the body to optimally recover for competition. The days and weeks after a main competition are characterized by low-intensity and low-volume training, with goals to induce regeneration and to prepare the athlete mentally and physically for the next training cycle (transition phase) [14, 16].

Although the concept of training periodization in elite endurance sports has been established for a long time, the coupling of periodized training with nutrition and body composition has gained scientific awareness only recently [17]. Stellingwerff’s group was one of the first to publish periodized nutrition guidelines for middle-distance athletes [17], they then expanded these recommendations for a multitude of power sports [18]. Nowadays, there are guidelines for carbohydrate, protein, and fat intake during training and competition phases, not exclusively focusing on endurance sports [19–21]. Meanwhile, for endurance athletes, sport-specific dietary intake recommendations were developed only for a few endurance disciplines (e.g., swimmers [22–25], distance runners [26], marathon/triathlon/road cycling [27]). But it remains unclear whether endurance athletes are actually following these nutrient guidelines across all seasonal training phases.

The validity of either body composition, energy intake, or TEE-determination in athletes strongly depends on the methods used. The measurement of body composition in general is prone to error. It has been shown that acute food or fluid ingestion [28], subject positioning [29], previous physical activity [30], and hydration status [31] have an impact on reliability of body composition measurement. Since endurance athletes often train several times per day, it might be difficult to assure best conditions for body composition assessment. According to a recent methodology review performed by Nana et al., only few of the studies, where body composition of athletes was measured with dual X-ray absorptiometry (DXA), provided details about their subject and device standardization [30]. However, other methods like skinfold measurements require highly experienced investigators [32] and strongly depend on the number of measurement sites and the formula used to calculate the percentage of body fat [33]. Therefore, it is important to report standardization protocols in order to evaluate the quality of data assessment. One main issue in assessing energy intake in athletes is the magnitude of under-reporting, which can amount to 10–45% of TEE [34]. It was shown that the magnitude of under-reporting increases as energy requirements increase [34]. Since endurance athletes are often characterized by high TEE, we must assume that these athletes are very prone to a high percentage of under-reporting. For determination of TEE objective methods such as doubly labelled water (DLW) or heart frequency measurements are available. However, in many studies subjective methods such as activity records and activity questionnaires are used in order to assess the activity level and TEE of subjects. These methods *estimate* TEE or activity level and their validity strongly depends on the breadth of the activity dimensions analyzed.

There exist some longitudinal studies that have assessed fluctuations in body composition, dietary intake, and/or TEE of endurance athletes across the training seasons [35–52], but no systematic reviews have been performed. Therefore, the purpose of this study was to (1) systematically analyze TEE, energy intake, and body composition in highly trained athletes of various endurance disciplines and of both sexes with focusing on objective assessment methods and (2) analyze fluctuations in these parameters across the training season. We hypothesized that endurance athletes show large fluctuations of TEE during different seasonal training phases due to differing exercise loads, and concomitant alterations in energy intake and body composition.

## Methods

The review protocol was developed according to the Meta-analysis of Observational Studies in Epidemiology Guidelines for meta-analyses and systematic reviews of observational studies [53].

### Search Strategy

A systematic literature search was performed to retrieve articles pertaining to body composition, energy intake, and TEE in endurance athletes across the training season. One researcher (JH) conducted the search for publications on 31 January 2015 in the electronic databases MEDLINE (via PubMed) and SPORTDiscus with Full Text (via EBSCOHost). A hand search of relevant reviews was performed to obtain additional articles missed by the database search. No individual or organization was contacted to receive further publications. To identify the population of endurance athletes, the following keywords connected with the Boolean operator “OR” were searched: endurance athletes, endurance-trained, endurance trained, aerobically trained, runners, swimmers, triathletes, skiers, cyclists, and rowers. To identify the outcome of body composition, TEE, and energy intake, the following keywords connected with the Boolean operator “OR” were searched: body composition, fat mass, fat-mass, fat free mass, fat-free mass, body fat, metabolic rate, energy expenditure, dietary intake, food intake, energy intake, food consumption, and macronutrient*. Terms for the study population and outcomes were combined by the use of the Boolean operator “AND”. Limits included articles published in the English language, human studies, and publishing date limits between 1990 and January 2015. Keywords were searched as free text in the title, abstract, and subject heading. A detailed overview of search strategies in the two databases can be obtained in Additional file 1: Table S1.

### Literature Selection

Two researchers independently assessed the eligibility of the records by screening the title, abstract, and keywords for inclusion and exclusion criteria. An agreement between the two researchers was quantified by kappa statistics [54]. The full texts of all abstracts meeting the eligibility criteria were retrieved and subjected to a second assessment for relevance performed by one author (JH).

The inclusion criteria included (1) articles reporting original data in peer-reviewed journals; (2) in vivo, human analyses; (3) adult endurance athletes (highly aerobically trained individuals who were engaged in a competitive endurance sport) with a mean age of 18–40 years; (4) reporting of training seasonal phase of data assessment; and (5) assessment of body composition and/or ad libitum daily energy intake and/or daily TEE. Articles were excluded from the review if (1) the article was only in abstract form or a case report, (2) data could not be split between the sexes (where both male and female subjects were analyzed), (3) body composition was assessed by skinfold measurements, (4) daily TEE was assessed by the use of questionnaires, and (5) descriptive quantitative results were not reported in a text or tabular form. Any difference in assessments between the two researchers was discussed in the first instance or resolved by a third author (KM).

### Methodological Quality Assessment

All relevant articles were examined for full methodological quality using a modified version of the Downs and Black [55] checklist for the assessment of the methodological quality of randomized and non-randomized studies of health care interventions. According to Fox et al. [56], 10 of the 27 criteria that logically applied to all of the types of studies included in this review were used. The maximum possible total score was 10. Two researchers assessed the study quality independently, with differences resolved by consensus or by a third author (KM). The agreement between the two researchers was quantified by kappa statistics [54]. Based on the assessment of the methodological study quality, no studies were excluded and no additional analyses were undertaken. The methodological quality of the included studies is shown in Additional file 2: Table S2.

### Data Extraction

Body composition, energy intake, and/or TEE data were extracted from all studies included in the review by the first author (JH). Demographic and methodological data were also extracted for the following confounding factors: age, sex, sports discipline, competition level, seasonal phase, and methods for assessing body composition, energy intake, and/or TEE. If the same subjects were analyzed during different time points in the same seasonal phase (e.g., energy intake before three different races, or assessment of energy intake at three time points during the training period), the first time point was chosen for data analysis to facilitate data entry and to avoid selection bias. If studies reported any intervention leading to a non-habitual behavior of athletes’ nutrient intakes (e.g., dietary supplementation), the baseline and/or control group data were used. To enable comparisons between studies, reported units were converted into standard units. These conversions were performed by using the reported mean values of the outcomes. Energy intake and TEE were reported in either absolute (kcal/day) or relative values (energy intake or TEE in relation to body weight [kcal/kg·day]). Body composition was converted into fat mass (%, kg) and fat-free mass (kg). According to the definition by Wang et al. [57], the terms lean body mass and fat-free mass (FFM) were considered synonymous. Duplicate publications from the same data set were identified according to the criteria published in the Cochrane Handbook for Systematic Reviews of Intervention [58]. The most complete record was then used for data extraction.

According to the traditional periodization model, the reported seasonal training phases of data assessment were clustered into three groups that included the preparation phase, the competition phase, and the transition phase [14–16]. A detailed overview of the clustering can be obtained in Table 1.

**Table 1:** Clustering of seasonal training phases for body composition, energy intake, and total energy expenditure

Preparation phase	Competition phase	Transition phase
Training/preparation/conditioning/peak training period Beginning/early/middle/ end of training season Beginning of season Before/pre-season High/low volume weeks Before/during/after high intensity/exhaustive training periods/training camps Intensified/overloaded/heavy training End of preparatory training phase Habitual/basic/normal training phase Non-competitive season	Before/during/after race/competition Taper phase Peak-season, in-season Top of performance Early/start/during/end of competitive season Pre-competition Mid/late season Beginning of competition preparatory period	Detraining Off-season Post-season After/between season Recreation Resting period

### Statistical Analysis

The main outcome measures were body composition (fat mass, FFM), energy intake, and TEE of endurance athletes across the season. Once all of the relevant data were extracted, the weighted mean and standard deviation of the weighted mean were calculated for the main outcome variables. Based on the number of subjects examined within the study, relative to the total number of subjects examined for the specific variable, a percentage weight (*w*) was allocated to each result within each outcome variable and used for the calculation of the overall weighted mean (*X̅*_*w*_) and standard deviation of the weighted mean (SD_*w*_) for each variable [59]. A capital “*N*” denotes the number of separate studies, while a small “*n*” denotes the number of included individual subjects.

Statistical analyses were performed using the statistical software SPSS statistics version 22 for Windows (IBM Corp., Chicago, IL, USA). *p* values < 0.05 were considered statistically significant. Kolmogorov-Smirnov tests were performed to check for normal distributions. All parameters were normally distributed except body mass, fat mass, and FFM. To test for comparisons of subgroups, one-factorial analyses of variance (ANOVAs) with Scheffé post hoc tests (parametric) and Kruskal-Wallis tests (*H*-test) with Mann-Whitney *U* post hoc tests (non-parametric) were performed. When multiple non-parametric post hoc tests were applied, Bonferroni-adjusted alpha levels were applied. Since parameters for body composition were not normally distributed, we abstained from multiple statistical comparisons between seasonal training phases and endurance disciplines to reduce the risk of type I errors. For comparisons of energy intake and TEE during different seasonal training phases, paired *t*-tests were used. The separate analysis of studies, where energy intake and TEE were assessed in parallel, and longitudinal studies that reported energy intake during different training season phases, were performed using the free software for meta-analysis Review Manager 5 version 5.3.5 for Windows (Cochrane Collaboration, Copenhagen, Denmark). The results were then presented as means and 95% confidence intervals (95% CI).

## Results

### Description of Studies and Assessment Methods

**Fig. 2:**
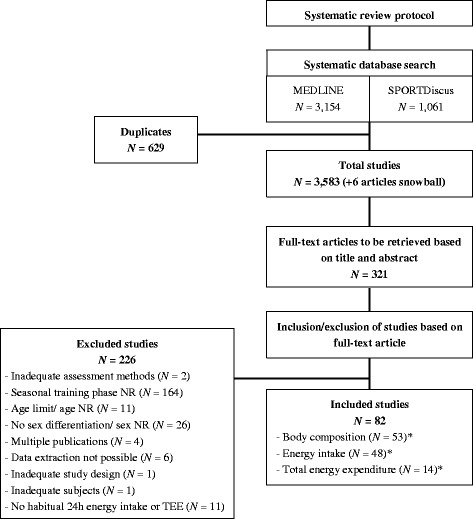
Flow chart for the present systematic review. NR = not reported. *Sum of studies not equal to total as multiple parameters were assessed in certain studies. *N* = number of studies

The flow chart for the study selection process is shown in Fig. 2. Data were extracted from 82 studies in endurance athletes, with 53 studies assessing body composition, 48 energy intake, and 14 TEE. The kappa value of 0.47 for the agreement between the two researchers who assessed the eligibility of records was considered to reflect a “fair agreement”, whereas “excellent agreement” (kappa value of 0.96) was obtained for the assessment of the methodological quality of included studies [54].

The characteristics of the included studies for body composition, energy intake, and TEE are shown in Table 2. In Additional file 3: Table S3, an overview of excluded studies and the reasons for their exclusion can be found.

**Table 2:** Characteristics of the studies included in the review of body composition (BC), energy intake (EI), and total energy expenditure (TEE)

Reference	Study design	*n* (sex)	Discipline (distance), level	Age (years)	Ethnicity, country	Assessment methods			Seasonal phase^a^	Quality rating
						BC	EI	TEE		
Armstrong et al. 2012 [80]	Observational study	42 (M)	Cyclists, nonelite	38 ± 6	NR, USA		24 h DR		2	8
Barr & Costill 1992 [43]	Observational study	24 (M)	Swimmers, tertiary	19.4 ± 0.4	NR, USA		2d DR		1, 2	8
Bemben et al. 2004 [35]	Observational study	11 (F)	Cross-country runners, tertiary	19.5 ± 0.4	NR, USA	DXA	3d DR		1, 3	8
Berg et al. 2008 [81]	Observational study	9 (M) 7 (F)	Athletes (UE), elite	27 [25–35] (M) 32 [26–42] (F)	NR, Sweden	BIA			2	8
Berg et al. 2008 [81]	Observational study	6 (M)	Athletes (UE), elite	27 [25–35]	NR, Sweden			HR	2	8
Bescós et al. 2012 [60]	Observational study	8 (M)	Cyclists (6), triathletes (2), non-professional	36.7 ± 4.7	NR, Spain		DR	HR	2	8
Boulay et al. 1994 [66]	Cross-sectional study	7 (M)	Cross-country skiers, provincial/national	21 ± 5	NR, Canada	UW	3d DR	HR	1	8
Brewer et al. 2013 [82]	RCT	9 (M)	Cyclists, NR	32.6 ± 7.4	NR, Australia	DXA			2	8
Brinkworth et al. 2002 [83]	RCT	6 (F)	Rowers, international	20.6 ± 2.3	NR, Australia		DR		2	7
Carbuhn et al. 2010 [36]	Observational study	16 (F)	Swimmers, tertiary	19 ± 1	NR, USA	DXA			1, 3	9
Costa et al. 2014 [63]	Cross-sectional study	19 (M) 6 (F)	Runners (UE), NR	39 ± 7	NR, UK		24 h recall	Accelerometry	2	8
Couzy et al. 1990 [44]	Observational study	6 (M)	Runners (MD), national/international	21.5 ± 0.7	NR, France		7d DR		1, 2	8
Decombaz et al. 1992 [84]	Observational study	17 (M)	Endurance skiers, NR	34.1 ± 1.4	NR, Switzerland		14d DR		1	8
Dellavalle & Haas 2014 [85]	RCT	28 (F)	Rowers, NR	19.8 ± 1.1 (PLA) 19.7 ± 0.9 (CON)	NR, USA		7d DR		1	8
Desgorces et al. 2004 [45]	Observational study	11 (M)	Rowers, NR	21.5 ± 0.8	NR, France		3d DR		1, 3	7
Desgorces et al. 2008 [86]	Observational study	13 (M)	Rowers, NR	21.5 ± 0.8	NR, France		3d DR		1	7
Drenowatz et al. 2012 [87]	Observational study	15 (M)	Endurance athletes (LD/UE), NR	23.6 ± 3.4	NR, USA	BodPod	FFQ		1	8
Drenowatz et al. 2013 [88]	Observational study	15 (M)	Endurance athletes, NR	23.6 ± 3.4	NR, USA			HR	1	8
Emhoff et al. 2013 [89]	Cross-sectional study	6 (M)	Cyclists/triathletes, competitive	24 ± 2	NR, USA		3d DR		2	8
Enqvist et al. 2010 [90]	Observational study	6 (M)	Endurance athletes (UE), NR	31 ± 4	NR, Sweden	BIA			2	8
Fudge et al. 2006 [11]	Observational study	9 (M)	Runners (MD/LD), national/international	21 ± 2	Kalenjin, Kenya	BIA	7d DR	DLW	1	8
Fudge et al. 2008 [91]	Cross-sectional study	14 (M)	Runners (MD/LD), national/international	22 ± 3	NR, Kenya	BIA	5d DR		2	8
Garcia-Roves et al. 1998 [92]	Cross-sectional study	10 (M)	Cyclists, international	27.6 ± 2.0	NR, Spain		3d DR		2	8
Garcia-Roves et al. 2000 [46]	Observational study	6 (M)	Cyclists, international	27.0 ± 1.9	NR, Spain		3d DR		1, 2	8
Gorsuch et al. 2013 [93]	RCT	10 (M) 10 (F)	Cross-country runners, tertiary	19.2 ± 0.4 (M) 19.9 ± 0.4 (F)	NR, USA	BodPod			3	8
Griffith et al. 1990 [94]	Observational study	6 (M)	Endurance athletes, NR	28	NR, USA	UW			1	8
Hassapidou & Manstrantoni 2001 [47]	Observational study	11 (F)	Runners (MD), regional	22.7 ± 2	NR, Greece		7d DR		1, 2	7
Hassapidou & Manstrantoni 2001 [47]	Observational study	9 (F)	Swimmers, regional	18.5 ± 1.1	NR, Greece		7d DR		1, 2	7
Havemann & Goedecke 2008 [95]	Observational study	45 (M)	Cyclists, NR	39 ± 10	NR, South Africa		3d DR		2	8
Heinonen et al. 1993 [96]	Cross-sectional study	30 (F)	Orienteers, NR	23.3 ± 3.1	NR, Finland	BIA			1	8
Heinonen et al. 1993 [96]	Cross-sectional study	29 (F)	Cyclists, NR	24.0 ± 5.7	NR, Finland	BIA			1	8
Heinonen et al. 1993 [96]	Cross-sectional study	28 (F)	Cross-country skiers, NR	21.3 ± 3.2	NR, Finland	BIA			1	8
Herring et al. 1992 [97]	Observational study	9 (F)	Endurance runners, NR	25.9 ± 2.4	NR, USA	UW	3d DR		1	9
Hill & Davies 2002 [69]	Cross-sectional study	7 (F)	Lightweight rowers, elite	20.0 ± 1.1	NR, Australia	DLW	4d DR	DLW	1	9
Hulton et al. 2010 [62]	Cross-sectional study	4 (M)	Cyclists (UE), non-professional	37 ± 4	NR, USA		6.5d DR	DLW	2	9
Jensen et al. 1992 [48]	Observational study	14 (M)	Cyclists, tertiary	23.1 ± 2.4	NR, USA		5d DR 3d DR		1, 2	7
Jones & Leitch 1993 [98]	Cross-sectional study	5 (M) 3 (F)	Swimmers, tertiary	19.8 (M) 20.7 (F)	NR, Canada	DLW			2	8
Jurimae et al. 1999 [99]	Cross-sectional study	10 (M)	Rowers, tertiary	21.6 ± 4.2	NR, Estonia	BIA			1	8
Jurimae et al. 2006 [100]	Cross-sectional study	8 (M)	Rowers, tertiary	21.5 ± 4.5	NR, Estonia	BIA			1	8
Jurimae & Jurimae 2004 [101]	Cross-sectional study	10 (F)	Rowers, tertiary	19.4 ± 1.6	NR, Estonia	DXA			2	8
Jurimae et al. 2007 [102]	Observational study	12 (M)	Rowers, national/international	20.8 ± 3	NR, Estonia	BIA			1	8
Jurimae et al. 2011 [103]	Cross-sectional study	9 (M)	Rowers, national	20.1 ± 1.6	NR, Estonia	DXA	3d DR		2	8
Kabasakalis et al. 2007 [37]	Observational study	4 (M)	Swimmers (sprint/MD), international	18.4 ± 1.2	NR, Greece	BIA			1, 2	8
Koshimizu et al. 2012 [104]	Cross-sectional study	24 (M)	Endurance athletes, elite	21.5 ± 3.4	NR, Japan	BodPod	3d DR		1	8
LaForgia et al. 1999 [38]	Observational study	16 (M)	Endurance athletes, NR	23.1 ± 4.7	NR, Australia	DXA			1, 3	8
Lazzer et al. 2012 [105]	Cross-sectional study	10 (M)	Runners (UE), amateur	38.2 ± 12.4	NR, Italy	BIA			2	8
Loftin et al. 1992 [39]	Observational study	5 (M) 5 (F)	Cross-country runners, tertiary	20.8 ± 1.1 (M) 20.8 ± 1.8 (F)	NR, USA	UW			2, 3	8
Maestu et al. 2010 [106]	Observational study	9 (M)	Rowers, international	19.7 ± 1.0	NR, Estonia	DXA			2	8
Magkos et al. 2007 [107]	Cross-sectional study	7 (M)	Endurance swimmers, national/international	19.4 ± 1.9	Caucasian, Greece	DXA			2	8
Magkos et al. 2007 [107]	Cross-sectional study	10 (M)	Endurance runners, national/international	23.4 ± 3.8	Caucasian, Greece	DXA			2	8
Maïmoun et al. 2003 [108]	Cross-sectional study	11 (M)	Cyclists, national	27.4 ± 5.8	NR, France	DXA			2	8
Maïmoun et al. 2003 [108]	Cross-sectional study	14 (M)	Triathletes, regional	25.7 ± 6.6	NR, France	DXA			2	8
Maïmoun et al. 2003 [108]	Cross-sectional study	13 (M)	Swimmers (sprint/MD), tertiary	25.4 ± 6.5	NR, France	DXA			2	8
Margaritis et al. 2003 [49]	Observational study	9 (M)	Triathletes (LD), NR	32.6 ± 10.5	NR, France		28d/14d DR		1, 2	8
Martin et al. 2002 [109]	Observational study	8 (F)	Cyclists, international	25.1 ± 4.0	NR, Australia		8–9d DR		2	8
Medelli et al. 2009 [110]	Cross-sectional study	23 (M)	Cyclists, international	28.5 ± 3.9	NR, France	DXA			1	7
Moses & Manore 1991 [111]	Observational study	17 (M)	Runners (LD), elite	25.7 ± 3.9	NR, USA		3d DR		2	8
Moses & Manore 1991 [111]	Observational study	9 (F)	Runners, NR	34.8 ± 6	NR, USA		3d DR		1	8
Motonaga et al. 2006 [112]	Cross-sectional study	6 (M)	Runners, sub-elite	19-21	NR, Japan	BIA		HR	1	8
Muoio et al. 1994 [113]	Cross-sectional study	6 (M)	Runners (LD), tertiary	21 ± 0.7	NR, USA	UW	4d DR		1	8
Noland et al. 2001 [40]	Observational study	12 (F)	Swimmers, tertiary	19.8 ± 0.1	NR, USA	UW			1, 2	7
Ousley-Pahnke et al. 2001 [114]	Cross-sectional study	15 (F)	Swimmers, tertiary	19.6 ± 1.2	NR, USA		4d DR		2	7
Palazzetti et al. 2004 [115]	Observational study	7 (M)	Triathletes, NR	32.9 ± 9.9	NR, France		28d DR		1	8
Palm et al. 2005 [116]	Cross-sectional study	11 (M)	Rowers, national	19.1 ± 3.8	NR, Estonia	DXA			2	8
Papadopoulou et al. 2012 [50]	Observational study	23 (M) 10 (F)	Cross-country skiers, international	20 ± 6 (M) 20 ± 5 (F)	NR, Greece	BIA	3d/1d DR		1 (BC/EI), 2 (EI)	8
Penteado et al. 2010 [117]	Cross-sectional study	31 (M)	Cyclists, NR	24.7 ± 3.2	NR, Brazil	DXA	4d DR		3	9
Peters & Goetzsche 1997 [51]	Observational study	151 (M) 22 (F)	Runners (UE), NR	37 ± 9.2 (M) 36 ± 6.1 (F)	NR, South Africa		24 h DR		1, 2	8
Phillips et al. 1993 [118]	Cross-sectional study	6 (M) 6 (F)	Runners, tertiary	23.3 ± 3.9 (M) 23.0 ± 4.9 (F)	NR, Canada	UW			1	8
Rehrer et al. 2010 [61]	Observational study	4 (M)	Cyclists, national/international	20 ± 3	NR, New Zealand	DXA	6d DR	DLW	2	8
Roberts & Smith 1992 [119]	Observational study	9 (M)	Swimmers, international	23 ± 2	NR, Canada		2d DR		1	8
Santos et al. 2014 [120]	Cross-sectional study	36 (M)	Swimmers, NR	19.1 ± 3.4 (M)	NR, Portugal	DXA			2	8
Santos et al. 2014 [120]	Cross-sectional study	38 (M) 10 (F)	Triathletes, NR	22.9 ± 5.4 (M) 20.4 ± 3.1 (F)	NR, Portugal	DXA			2	8
Santos et al. 2014 [120]	Cross-sectional study	11 (M) 16 (F)	Athletic athletes, NR	20.1 ± 3.0 (M) 21.3 ± 4.1 (F)	NR, Portugal	DXA			2	8
Sato et al. 2011 [121]	Observational study	6 (M) 13 (F)	Swimmers, tertiary	19.5 ± 1.0 (M) 19.4 ± 1.0 (F)	NR, Japan	BIA	3d DR		1	9
Schena et al. 1995 [122]	Cross-sectional study	73 (M)	Cross-country skiers, NR	26.9 ± 4.4	NR, Italian		7d DR		1	8
Schena et al. 1995 [122]	Cross-sectional study	33 (M)	Roller skiers, NR	25.6 ± 4.1	NR, Italian		7d DR		1	8
Schena et al. 1995 [122]	Cross-sectional study	35 (M)	Runners, NR	26.8 ± 3.7	NR, Italian		7d DR		1	8
Schena et al. 1995 [122]	Cross-sectional study	18 (M)	Cyclists, NR	30.1 ± 5.1	NR, Italian		7d DR		1	8
Schenk et al. 2010 [123]	Cross-sectional study	25 (M)	Mountain bikers, amateur	38 ± 10	NR, Austria	BIA			2	8
Schulz et al. 1992 [68]	Cross-sectional study	9 (F)	Runners (LD), national/international	26.0 ± 3.3	NR, USA	UW	6d DR	DLW	1	8
Sherman et al. 1993 [124]	Cross-sectional study	18 (M)	Cyclists, NR	30 ± 3 (*n* = 9) 25 ± 3 (*n* = 9)	NR, USA	UW			1	7
Sherman et al. 1993 [124]	Cross-sectional study	18 (M)	Runners, NR	30 ± 3 (*n* = 9) 34 ± 3 (*n* = 9)	NR, USA	UW			1	7
Siders et al. 1991 [41]	Observational study	6 (M) 11 (F)	Swimmers, tertiary	19.5 ± 1.0 (M) 19.2 ± 1.0 (F)	NR, USA	UW			1, 2	8
Siders et al. 1993 [42]	Observational study	31 (M) 43 (F)	Swimmers (sprint), tertiary	20.5 ± 1.9 (M) 19.7 ± 1.4 (F)	NR, USA	UW			1, 2	8
Simsch et al. 2002 [125]	Cross-sectional study	6 (M)	Rowers, NR	18.7	NR, Germany	Near infrared			1	7
Sjodin et al. 1994 [67]	Cross-sectional study	4 (M) 4 (F)	Cross-country skiers, international	26 ± 2 (M) 25 ± 2 (F)	NR, Sweden	DLW	4d DR (M) 5d DR (F)	DLW	1	8
Sundby & Gorelick 2014 [126]	Cross-sectional study	10 (F)	Runners, tertiary	25.7 ± 4.7	NR, USA	BodPod			1	8
Taylor et al. 1997 [52]	Observational study	7 (F)	Swimmers, national	19 ± 2	NR, South Africa		7d DR		1, 2	8
Tomten & Hostmark 2006 [127]	Cross-sectional study	20 (F)	Runners, recreational/national	34.8 ± 1.7 (R) 26.0 ± 1.8 (IR)	Caucasian, Norway	DXA	3d DR		2	8
Trappe et al. 1997 [70]	Cross-sectional study	5 (F)	Swimmers, international	19 ± 1	NR, USA		2d DR	DLW	1	8
Vaiksaar et al. 2011 [128]	Observational study	11 (F)	Rowers, national	18.4 ± 1.9	Caucasian, Estonia	DXA	3d DR		1	8
Winters et al. 1996 [71]	Cross-sectional study	10 (F)	Runners (LD), tertiary	19.7 ± 1.7	Caucasian, USA	UW	3d DR	HR	2	8
Witard et al. 2011 [129]	Cross-sectional study	8 (M)	Cyclists, NR	27 ± 8	NR, UK		3d DR		1	8
Yeater et al. 1996 [130]	Cross-sectional study	8 (M)	Cross-country runners, tertiary	21 [18–30]	NR, USA	UW			1	8
Zajac et al. 2014 [131]	Observational study	8 (M)	Cyclists, NR	28.3 ± 3.9	NR, Poland	BIA			1	8
Zalcman et al. 2007 [132]	Cross-sectional study	18 (M) 6 (F)	Adventure racers, national/international	30.9 ± 5.8 (M) 30.3 ± 7.8 (F)	NR, Brazil	BodPod	3d DR		1	8

*Note. * Age is given as *M ± SD* or *M* [range]

*F* female, *M* male, *UE* ultra-endurance, *MD* middle distance, *LD* long distance, *NR* not reported, *RCT* Randomized Controlled Trial, *R* regular menstrual function, *IR* irregular menstrual function, *PLA* placebo group, *CON* control group, *DXA* dual-energy X-ray absorptiometry, *BIA* bioelectrical impedance analysis, *UW* underwater/hydrostatic weighing, *DR* dietary record, *FFQ* Food Frequency Questionnaire, *HR* heart rate monitoring, *DLW* doubly labelled water

^a^(1) = preparation phase, (2) = competition phase, (3) = transition phase

The cumulative number of subjects included in the analysis was 1674 (71.4% male). Runners (27.8%), cyclists (18.7%), and swimmers (16.4%) comprised the largest proportion of subjects. All athletes for whom an endurance sports discipline was not described or for whom multiple endurance disciplines were mentioned were grouped into “other endurance athletes” (13.5%). On average, the mean age, VO_2max_, and training volume of study estimates were 26.3 ± 6.7 years, 61.8 ± 6.0 mL/kg min, and 12.0 ± 6.9 h/week, respectively (*X̅*_*w*_ ± SD_*w*_). A detailed overview of physical characteristics of included study estimates is shown in Table 3.

**Table 3:** Physical characteristics of included study estimates

Endurance discipline (*N*)	*n*	Age [years]	Height [cm]	Body mass [kg]	BMI [kg/m^2^]	VO_2_max [mL/kg min]	Train load [h/week]^b^
Cyclists							
Total (18)	313	30.9 ± 6.1	177 ± 5	75.4 ± 5.9	23.4 ± 1.6	62.4 ± 6.2	14.0 ± 8.5
Male (16)	276	31.8 ± 5.6	179 ± 3	74.4 ± 5.5	23.6 ± 1.6	65.0 ± 4.8	15.2 ± 9.6
Female (2)	37	24.2 ± 0.5	166 ± 1	61.2 ± 1.1	22.1 ± 0.6	55.8 ± 4.0	–
Runners							
Total (23)^a^	465	30.3 ± 7.1	172 ± 5	64.1 ± 7.4	20.3 ± 1.3	61.7 ± 7.2	8.6 ± 4.2
Male (16)	330	31.4 ± 6.9	175 ± 3	67.9 ± 5.5	20.6 ± 1.4	64.3 ± 6.7	8.6 ± 4.3
Female (13)	135	27.4 ± 6.7	167 ± 3	55.6 ± 2.2	19.9 ± 1.0	57.3 ± 5.8	8.7 ± 4.0
Swimmers							
Total (16)^a^	275	19.9 ± 1.5	176 ± 6	69.5 ± 5.9	22.4 ± 0.7	–	17.2 ± 10.3
Male (10)	141	20.3 ± 1.9	181 ± 3	74.3 ± 3.2	22.7 ± 0.7	–	13.4 ± 5.6
Female (10)	134	19.4 ± 0.4	170 ± 4	63.9 ± 2.5	22.0 ± 0.5	–	23.1 ± 12.8
Rowers							
Total (14)	151	20.2 ± 1.0	180 ± 9	76.1 ± 10.3	23.5 ± 1.0	54.6 ± 8.5	7.2 ± 2.4
Male (9)	89	20.6 ± 1.0	188 ± 3	85.4 ± 5.0	24.0 ± 0.9	–	7.2 ± 2.4
Female (5)	62	19.6 ± 0.6	171 ± 2	66.3 ± 2.2	22.9 ± 0.7	–	–
Cross-country skiers							
Total (6)^a^	166	25.0 ± 4.3	175 ± 5	65.9 ± 4.5	21.5 ± 0.7	61.9 ± 4.3	11.5 ± 0.5
Male (5)	124	26.2 ± 4.2	177 ± 2	68.1 ± 1.4	21.7 ± 0.6	–	11.7 ± 0.4
Female (3)	42	21.3 ± 1.3	168 ± 2	59.2 ± 3.5	21.0 ± 0.8	–	–
Triathletes							
Total (4)^a^	78	25.1 ± 4.2	175 ± 3	66.2 ± 3.6	21.6 ± 0.7	65.3 ± 0.4	11.4 ± 2.0
Male (4)	68	25.8 ± 4.0	176 ± 0	67.5 ± 1.8	21.8 ± 0.5	65.3 ± 0.4	11.6 ± 2.1
Female (1)	10	–	–	–	–	–	–
Other endurance athletes							
Total (13)^a^	226	25.2 ± 4.0	176 ± 6	69.1 ± 6.7	22.5 ± 1.1	61.7 ± 4.7	10.5 ± 3.8
Male (12)	167	25.5 ± 4.0	178 ± 3	72.7 ± 3.4	22.9 ± 0.9	63.8 ± 3.8	11.2 ± 4.5
Female (4)	59	24.5 ± 3.7	168 ± 1	59.3 ± 1.8	21.3 ± 0.6	56.8 ± 2.3	9.1 ± 0.7
Total							
Total (82)^a^	1674	26.3 ± 6.7	176 ± 6	68.7 ± 8.0	22.2 ± 1.5	61.8 ± 6.0	12.0 ± 6.9
Male (63)	1195	27.7 ± 6.8	179 ± 4	72.1 ± 6.5	22.6 ± 1.5	64.4 ± 4.8	11.6 ± 5.6
Female (34)	479	22.9 ± 5.1	169 ± 3	60.5 ± 4.5	21.4 ± 1.2	56.6 ± 4.6	12.8 ± 9.0

*Note*. Data are shown in weighted mean and standard deviation of the weighted mean (X̅_w_ ± SD_w_)

*N* = number of studies, *n* = cumulative number of subjects, *BMI* body mass index, – = insufficient data

^a^Sum of male and female studies not equal to total as in certain studies both sexes were assessed

^b^Calculated as the following: 1 h of training = 25 km cycling or 10 km running or 2 km swimming

Body composition was assessed by DXA in 32.1% of studies, by bioelectrical impedance analysis (BIA) in 25.6% of studies, and by hydrostatic weighing in 25.6% of studies. In 71.7% of the studies, where body composition was measured, no details of standardization were provided. Ten studies (18.9%) reported some standardization details, whereas only three studies (5.7%) reported satisfactory details about their standardization. For determination of energy intake, dietary records (95.1%) with a mean observation time of 4.7 ± 4.1 days were most often utilized. Dietary recall (3.3%) and food frequency questionnaires (FFQs, 1.6%) played secondary roles in energy intake assessments. Half of the studies (50.0%) used DLW for determination of TEE. Other methods included heart rate monitoring (33.3%) and accelerometers (16.7%). The studies using heart rate monitoring for estimation of TEE used individual derived linear relationships between heart rate and oxygen consumption (HR–VO_2_) during different tasks to estimate the oxygen cost and energy expenditure during the observation period. Two third of the studies used the 24-h heart rate recordings and the individual HR–VO_2_ relationship to estimate TEE (gross calculation). Two studies calculated TEE by summation of activity energy expenditure (based on individual HR–VO_2_ relationship) and resting metabolic rate (RMR; net calculation).

### Total Energy Expenditure and Energy Intake

In total, 14 studies where TEE was assessed during various seasonal training phases were identified by the literature search. Since no study assessed TEE during the transition phase, only data during the preparation phase (*N* = 8) and the competition phase (*N* = 6) are shown. In addition, due to limited data, no separations between the sexes and endurance disciplines of TEE were performed.

Absolute and relative TEE were significantly higher during the competition phase than during the preparation phase (9869 ± 4129 vs. 4345 ± 1062 kcal/day, and 98.9 ± 46.5 vs. 68.5 ± 11.4 kcal/kg·day, respectively, all *p* < 0.001). Most of the studies assessing TEE during the competitive phase were conducted during an ultra-endurance competition (*N* = 5), such as during a 24-h team relay cycling race [60], during a 6-day cycling stage race [61], or during a 4851-km team relay cycling race [62]. The maximum TEE amounted to 13,862 kcal/day and 156.0 kcal/kg·day, respectively, observed in male ultra-endurance runners during a 24-h ultra-marathon [63]. The absolute and relative TEE were significantly higher than the energy intake in the preparation phase (4345 ± 1062 vs. 2915 ± 761 kcal/day, and 68.5 ± 11.4 vs. 42.8 ± 10.5 kcal/kg·day, respectively, all *p* < 0.001) and competition phase (9869 ± 4129 vs. 3156 ± 967 kcal/day, and 98.9 ± 46.5 vs. 43.5 ± 11.3 kcal/kg·day, respectively, all *p* < 0.001).

**Fig. 3:**
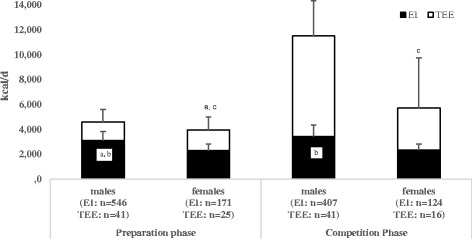
Energy intake (EI) and total energy expenditure (TEE) in kcal/day of endurance athletes. Data are shown in weighted mean and standard deviation of the weighted mean (X̅_w_ ± SD_w_). *n* = number of cumulative subjects

**Fig. 4:**
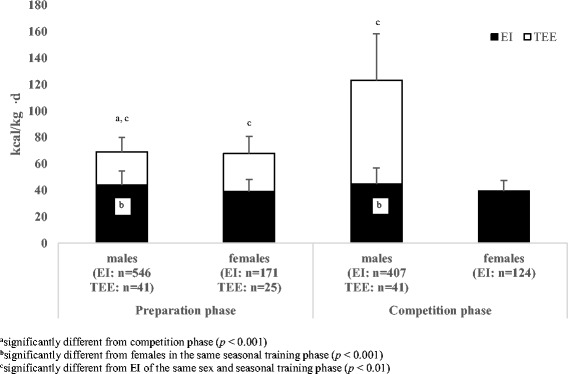
Energy intake (EI) and total energy expenditure (TEE) in kcal/kg·day of endurance athletes. Data are shown in weighted mean and standard deviation of the weighted mean (X̅_w_ ± SD_w_). *n* = number of cumulative subjects. No data for TEE of females during competition phase available

Absolute and relative energy intake was higher in males compared to females in the preparation phase (3111 ± 717 vs. 2291 ± 525 kcal/day, and 44.0 ± 10.6 vs. 39.0 ± 9.1 kcal/kg·day, respectively, all *p* < 0.001) and competition phase (3405 ± 940 vs. 2337 ± 483 kcal/day, and 44.8 ± 11.9 vs. 39.3 ± 7.9 kcal/kg·day, respectively, all *p* < 0.001, Figs. 3 and 4).

In males, the absolute energy intake was higher during the competition phase compared to the preparation phase (*p* < 0.001), whereas relative energy intake was unchanged (*p* = 0.553). In females, neither the absolute (*p* = 0.735) nor relative (*p* = 0.951) energy intake was different between the two seasonal training phases.

Table 4 provides a detailed overview of the absolute and relative energy intakes differentiated by sex, endurance discipline, and seasonal training phase. Energy intake was significantly higher in male runners, swimmers, and rowers compared to their female counterparts during both the preparation and competition phases (all *p* < 0.01). In male and female runners, male endurance athletes, and combined male and female rowers and cross-country skiers, the energy intake was higher during the competition phase compared to the preparation phase, whereas for male and female swimmers, energy intake was higher during the preparation phase (all *p* < 0.01). The energy intake of female runners and rowers during the preparation phase was significantly lower than that of all other endurance athletes (all *p* < 0.05). Reasons for the lower energy intake in female rowers might be that during preparation phase the athletes often reduce their energy intake in order to reduce concomitantly their body weight to start in the lightweight category. During pre-season, body mass may reduce by as much as 8% among lightweight rowers [64]. Runners, in general, profit from a low body mass since greater economy of movement and better thermoregulatory capacity from a favorable ratio of weight to surface area and less insulation from subcutaneous fat tissue is reached [10].

**Table 4:** Energy intake in kcal/day and kcal/kg/day of endurance athletes in preparation and competition phase

	Preparation			Competition		
Endurance discipline	*n*	Energy intake [kcal/day]	Energy intake [kcal/kg·day]	*n*	Energy intake [kcal/day]	Energy intake [kcal/kg·day]
Cyclists						
Total	46	3789 ± 764^d,e,f^	52.3 ± 13.3^d,e^	133	3600 ± 1102^d^	46.9 ± 17.7^d,f^
Male	46	3789 ± 764^d,e^	52.3 ± 13.3^d,e^	125	3603 ± 1137	45.9 ± 18.0
Female	–	–	–	–	–	–
Runners						
Total	278	2489 ± 425^a^	38.2 ± 7.8^a^	272	3042 ± 788	42.7 ± 4.7
Male	207	2640 ± 366^a,b,f^	38.3 ± 8.6^a^	203	3298 ± 713^b^	43.8 ± 3.2^b^
Female	71	2046 ± 230^a^	38.0 ± 4.6^c^	69	2291 ± 443	39.4 ± 6.4
Swimmers						
Total	73	3366 ± 902^a,d,e,g^	48.7 ± 9.6^a,d,e^	55	2769 ± 681^g,h^	40.1 ± 7.7^g^
Male	39	3963 ± 762^a,b^	53.2 ± 9.5^a,b,d,e^	24	3462 ± 341^b^	46.2 ± 6.5^b^
Female	34	2683 ± 450^a,d,e^	43.6 ± 6.9^a,e^	31	2234 ± 256	35.4 ± 4.7
Rowers						
Total	70	2426 ± 448^a^	33.9 ± 4.5^a^	15	3633 ± 1097	46.8 ± 10.9
Male	24	2921 ± 326^b,f^	36.0 ± 0.1^b^	–	–	–
Female	46	2168 ± 330	32.8 ± 5.2^c^	–	–	–
Cross-country skiers						
Total	138	3224 ± 917^a,d,e,g^	48.3 ± 12.7^a,d,e^	33	2091 ± 53.2^d,e,f,g^	32.7 ± 2.9^c^
Male	124	3287 ± 876^d,f,g^	48.3 ± 11.6^d,e^	–	–	–
Female	14	2663 ± 1107^d,e^	49.1 ± 20.3	–	–	–
Triathletes						
Total	16	3162 ± 159^d,e^	45.7 ± 2.6^e^	–	–	–
Male	16	3162 ± 159^f,g^	45.7 ± 2.6	–	–	–
Female	–	–	–	–	–	–
Other endurance athletes						
Total	96	3261 ± 282^a,d,e,g^	46.5 ± 5.1^a,d,e^	14	4656 ± 1070	–
Male	90	3274 ± 286^a,d,f,g^	46.3 ± 5.2^a,d,e,f^	14	^d,f,g,h^	–
Female	–	–	–	–	4656 ± 1070^c^ –	–
Total						
Total	717	2915 ± 761^a^	42.8 ± 10.5	531	3156 ± 967	43.5 ± 11.3
Male	546	3111 ± 717^a,b^	44.0 ± 10.6^b^	407	3405 ± 940^b^	44.8 ± 11.9^b^
Female	171	2291 ± 525	39.0 ± 9.1	124	2337 ± 483	39.3 ± 7.9

*Note*. Data are shown in weighted mean and standard deviation of the weighted mean (X̅_w_ ± SD_w_)

*n* = cumulative number of subjects, – = insufficient data

^a^Significantly different from athletes of the same endurance discipline and sex during competition phase (*p* < 0.01)

^b^Significantly different from females of the same endurance discipline and seasonal training phase (*p* < 0.01)

^c^Significantly different from all other endurance disciplines of the same sex and seasonal training phase (*p* < 0.05)

^d^Significantly different to runners of the same sex and seasonal training phase (*p* < 0.05)

^e^Significantly different to rowers of the same sex and seasonal training phase (*p* < 0.05)

^f^Significantly different to swimmers of the same sex and seasonal training phase (*p* < 0.05)

^g^Significantly different to cyclists of the same sex and seasonal training phase (*p* < 0.05)

^h^Significantly different to cross-country skiers of the same sex and seasonal training phase (*p* < 0.05)

**Fig. 5:**
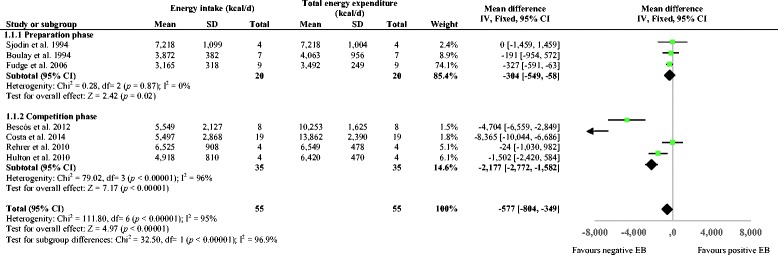
Energy balance (EB) of male endurance athletes during preparation and competition phase

**Fig. 6:**
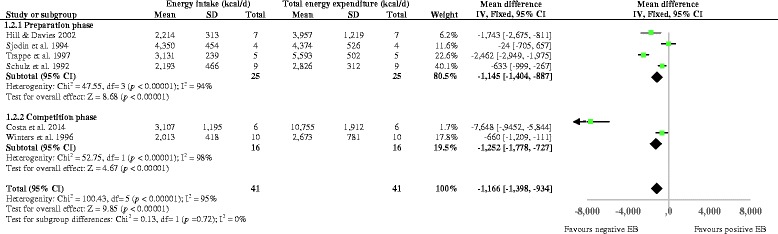
Energy balance (EB) of female endurance athletes during preparation and competition phase

**Fig. 7:**
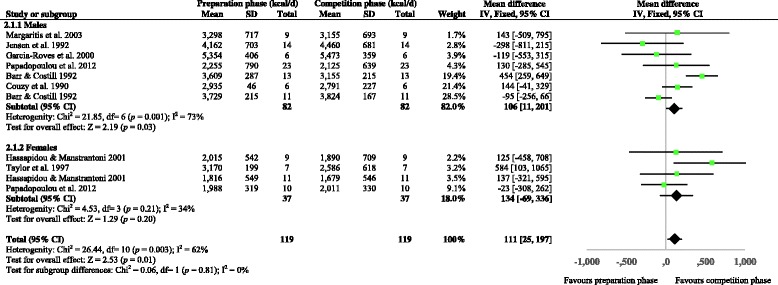
Forest plot for comparison of energy intake during preparation and competition phase in endurance athletes

A separate analysis of energy balance was performed by including only studies where both energy intake and expenditure were assessed in parallel. Male endurance athletes showed a significant energy deficit of 304 kcal/day (95% CI −549, −58, *p* = 0.02) during the preparation phase and 2177 kcal/day (95% CI −2772, −1582, *p* < 0.0001) during the competition phase (Fig. 5). In female endurance athletes, a negative energy balance was also observed during the preparation phase (−1145 kcal/day, 95% CI −1404, −887, *p* < 0.0001) and the competition phase (−1252 kcal/day, 95% CI −1778, −727, *p* < 0.0001, Fig. 6). The relative energy deficit was 6.6% of TEE during the preparation phase and 18.9% during the competition phase in males, and 29.0% of TEE during the preparation phase and 22.0% during the competition phase in females. When comparing energy intake during the preparation and competition phases by solely including studies where energy intake was assessed in both phases (*N* = 8), the energy intake was higher during the competition phase, being significant in males (+106 kcal/day, *p* = 0.03), but not in female endurance athletes (+134 kcal/day, *p* = 0.20, Fig. 7).

In more than half (53.7%) of the female study populations, where TEE was assessed, the menstrual status was not reported. 24.4% of the female study populations were eumenorrheic, whereas in 22.0% menstrual irregularities were reported. However, a separate statistical analysis assessing seasonal training phase differences of TEE between eumenorrheic and amenorrheic athletes could not be performed, since the cumulative number of subjects was too low in the single training phases.

### Body Composition

**Fig. 8:**
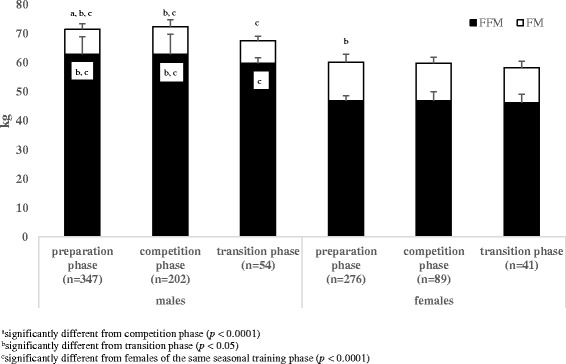
Fat-free mass and fat mass of endurance athletes during preparation, competition, and transition phase. Data are shown in weighted mean and standard deviation of the weighted mean (X̅_w_ ± SD_w_). *n* = number of cumulative subjects

For the total sample during the competition phase, both body mass and FFM were significantly higher compared to the preparation and transition phases (*p* < 0.05, Table 5). For the percentage of fat mass, no differences were detected between the seasonal training phases (*p* > 0.05). Since the percentage of female data on total data varies between the seasonal training phases, we further split the data by sex. In males, the body mass was lowest during the transition phase (*p* < 0.05) and absolute and relative fat mass were highest during the competition phase (all *p* < 0.05). FFM was lowest during the transition phase (*p* < 0.001, Fig. 8). For females, absolute and relative body fat were higher during the preparation phase compared to those during the transition phase (*p* < 0.01, Fig. 8). Neither body mass nor FFM differences between seasonal training phases were observed (all *p* > 0.05). When separately analyzing the few studies where body mass and composition were assessed during both the preparation and competition phases (*N* = 5), male and female endurance athletes showed a significantly lower percentage of body fat and higher absolute FFM during the competition phase compared to the preparation phase (18.2 ± 5.0% vs. 19.6 ± 5.0%, and 56.6 ± 8.7 kg vs. 54.0 ± 8.7 kg, respectively, all *p* < 0.0001).

**Table 5:** Body composition of included study estimates across the season

	Preparation				Competition				Transition			
Endurance discipline	*n*	Body mass [kg]	Body fat [%]	Fat-free mass [kg]	*n*	Body mass [kg]	Body fat [%]	Fat-free mass [kg]	*n*	Body mass [kg]	Body fat [%]	Fat-free mass [kg]
Cyclists ^a^												
Total	60	67.8 ± 6.5	16.7 ± 6.8	55.4 ± 9.2	49	75.3 ± 3.3	15.1 ± 1.3	62.5 ± 4.7	–	–	–	–
Male	31	73.3 ± 4.2	11.6 ± 1.7	64.1 ± 2.7	49	75.3 ± 3.3	15.1 ± 1.3	62.5 ± 4.7	–	–	–	–
Female	–	–	–	–	–	–	–	–	–	–	–	–
Runners ^a^												
Total	77	58.0 ± 5.7	12.5 ± 4.5	50.7 ± 7.2	74	60.7 ± 6.4	14.5 ± 5.2	50.4 ± 6.8	40	58.4 ± 5.3	15.6 ± 4.7	49.4 ± 7.3
Male	35	62.3 ± 5.3	9.2 ± 2.4	57.1 ± 5.8	39	63.4 ± 7.8	10.3 ± 3.6	55.7 ± 4.6	15	64.8 ± 2.1	9.6 ± 0.9	58.5 ± 2.5
Female	42	54.4 ± 2.6	16.7 ± 2.7	45.3 ± 1.7	35	57.7 ± 1.5	19.2 ± 0.7	44.4 ± 2.3	25	54.5 ± 1.4	19.1 ± 0.5	44.0 ± 1.0
Swimmers ^a^												
Total	166	69.1 ± 6.0	18.3 ± 5.6	54.8 ± 8.0	93	69.9 ± 6.5	16.0 ± 5.0	57.5 ± 8.2	–	–	–	–
Male	83	73.5 ± 2.7	12.9 ± 1.3	63.1 ± 2.4	56	75.5 ± 2.8	12.2 ± 1.2	64.4 ± 4.7	–	–	–	–
Female	83	63.5 ± 2.5	23.7 ± 1.4	47.6 ± 1.1	37	63.5 ± 2.2	21.8 ± 2.2	49.5 ± 1.3	–	–	–	–
Rowers ^a^												
Total	54	78.1 ± 10.7	16.1 ± 7.1	65.8 ± 13.8	39	80.7 ± 10.1	14.3 ± 6.5	66.0 ± 12.2	–	–	–	–
Male	36	84.7 ± 5.6	11.3 ± 1.1	75.1 ± 4.9	29	86.2 ± 4.0	10.5 ± 1.0	72.9 ± 3.4	–	–	–	–
Female	18	64.8 ± 3.2	25.8 ± 2.5	47.4 ± 0.4	–	–	–	–	–	–	–	–
Cross-country skiers ^a^												
Total	76	63.7 ± 5.9	15.7 ± 5.7	53.9 ± 7.7	–	–	–	–	–	–	–	–
Male	34	69.3 ± 2.3	10.3 ± 1.6	62.2 ± 1.7	–	–	–	–	–	–	–	–
Female	42	59.2 ± 3.5	20.1 ± 3.6	47.1 ± 0.9	–	–	–	–	–	–	–	–
Triathletes ^a^												
Total	48	64.2 ± 3.3	13.6 ± 3.3	54.8 ± 5.2	–	–	–	–	–	–	–	–
Male	–	–	–	–	–	–	–	–	–	–	–	–
Female	–	–	–	–	–	–	–	–	–	–	–	–
Other endurance athletes ^a^												
Total	142	67.9 ± 6.8	15.7 ± 4.2	57.5 ± 8.0	22	71.8 ± 11.0	18.5 ± 2.5	58.8 ± 10.6	–	–	–	–
Male	90	72.6 ± 3.2	13.0 ± 2.7	62.8 ± 4.3	15	79.2 ± 0.2	16.8 ± 0.2	65.8 ± 0.3	–	–	–	–
Female	52	59.8 ± 1.5	20.3 ± 1.6	48.2 ± 2.0	–	–	–	–	–	–	–	–
Total												
Total	623	67.5 ± 7.1^b^	15.9 ± 5.7	55.8 ± 9.2^b^	291	70.8 ± 8.6	15.2 ± 4.8	57.6 ± 9.5	95	65.3 ± 7.1^b^	15.1 ± 4.8	54.0 ± 7.2^b^
Male	347	72.0 ± 6.7^b,c^	11.8 ± 2.3^b,c^	63.0 ± 5.9^c^	202	74.5 ± 8.1	12.6 ± 2.8	62.9 ± 6.9	54	69.7 ± 3.4^b^	11.2 ± 1.7^b^	59.8 ± 1.9^b^
Female	276	60.5 ± 4.1	21.6 ± 3.6^c^	47.0 ± 1.6	89	60.2 ± 4.4	21.2 ± 2.4	47.0 ± 3.0	41	59.4 ± 6.4	20.2 ± 1.4	46.2 ± 2.9

*Note*. Data are shown in weighted mean and standard deviation of the weighted mean (X̅_w_ ± SD_w_)

*n =* cumulative number of subjects, *–* = insufficient data

^a^Data not normal distributed. To limit the risk of type I error no statistical comparison between seasonal training phases differentiated by sex and endurance discipline were performed

^b^Significantly different from competition phase (*p* < 0.05)

^c^Significantly different from transition phase (*p* < 0.05)

In more than one third (34.5%) of the female study populations, where body composition was assessed, the menstrual status was not reported. 39.7% of the female study populations were eumenorrheic, whereas 16.4% menstrual irregularities were reported. However, a separate analysis between eumenorrheic and amenorrheic athletes could not be performed, since the cumulative number of subjects during the different seasonal training phases was too low.

## Discussion

In this systematic review, we examined fluctuations in TEE, energy intake, and/or body composition in endurance athletes across the training season. We found that some, but not all, of the investigated outcomes depended on the time point of data assessment during seasonal training. TEE was highest during the competition phase and higher than energy intake in all seasonal training phases. Alterations in TEE did not lead to adaptations of energy intake in females, whereas in males, a higher absolute energy intake during the competition phase was observed. The finding that male endurance athletes demonstrated the highest fat mass values during the competition phase and the lowest FFM during the transition phase seems to be an anomaly from the pooling of data.

Our systematic search initially yielded many studies where TEE, energy intake, or body composition in endurance athletes were investigated. Only a few (2%) reported the time point of data collection with regard to the training season and could thus be included in this review. This is unfortunate since our analysis clearly illustrates how training volume and related TEE vary importantly with seasonal training phases. Specifically and expectedly, both absolute and relative TEEs were significantly higher during the competition phase compared to the preparation phase. Interestingly, these differences were only partly in agreement with alterations in energy intake and/or body composition of endurance athletes.

During the transition phase, limited data for TEE and energy intake of endurance athletes was available. Only for body composition, it was possible to compare with other seasonal training phases, although the number of study estimates and therefore, explanatory power, was weak. Future research on elite athletes should focus on the effects of a sudden stop or reduction in TEE on body composition (e.g., because of injury). There exist only a few studies (with conflicting results) where this question has been examined. Ormsbee and Arciero investigated the effects of 5 weeks of detraining on body composition and RMR in eight male and female swimmers [65]. RMR decreased, whereas fat mass and body weight increased with detraining. In contrast, LaForgia et al. showed that after 3 weeks of detraining, no differences in RMR and percentage of fat mass occurred in male endurance athletes [38]. Unfortunately, energy intake was not reported in either of these studies. Thus, it remains unclear when, whether, and to what extent the body adapts (through changes in energy intake and/or body composition) for the decrease in TEE caused by detraining.

Our analysis highlights an important apparent negative energy balance in endurance athletes, both in the preparation and competition phases, when separately examining the energy balance in articles where both energy intake and TEE were assessed (*N* = 11). Negative energy balance was reported during the preparation phase in male [66, 67] and female [67] cross-country skiers, male [11] and female [68] runners, and female lightweight rowers [69] and swimmers [70], and amounted to a mean of 304 kcal/day (4.7% of TEE) for males and 1145 kcal/day (27.8%) for females. During the competition phase, a negative energy balance was reported in male cyclists and triathletes [60], male [63] and female [63, 71] runners, and male cyclists [61, 62], averaging 2177 kcal/day (32.5%) for male and 1252 kcal/day (47.9%) for female endurance athletes. The most obvious explanation for these energy deficits is likely the classical issue of under-reporting energy intake through self-assessment in human studies. A review of nine studies using DLW to validate self-reported energy intake in athletes revealed that under-reporting can amount to 10–45% of TEE [34]. Since under-reporting increases in magnitude as energy requirements increase [34], we must assume that under-reporting in the present study estimates was more important during the competition phase. Even when 45% was added to the energy intake of all athletes included in our review, there still remained a negative energy balance of 118 kcal (2.7% of TEE) in the preparation and 5293 kcal (53.6%) in the competition phase. Another explanation for the negative energy balance might be the low accuracy and precision of methods used to estimate energy intake in athletes in the articles included in our review. For example, mostly dietary records with a mean observation time of 4.7 ± 4.1 days were used. According to Magkos and Yannakoulia, for athletes, a 3–7-day diet-monitoring period would be enough for reasonably accurate and precise estimations of habitual energy and macronutrient consumption [34]. However, other methods like FFQs and dietary recalls were also used for energy intake estimations. These methods are both memory-dependent and show lower accuracy and precision than prospective methods like dietary records [72]. However, even when only articles were considered where energy intake was assessed by the use of dietary records, the error remained high (2.5% of TEE during the preparation phase and 54.9% during the competition phase). Finally, the high negative energy balance during the competition phase may also be explained by the fact that, apart from one study, all included studies investigated the TEE during the days with actual competition and not during habitual training days in the competition phase. Thus, it is likely that the TEE during this phase was over-estimated. During the preparation phase, a negative energy balance leading to increased energy store utilization might be desirable by coaches and athletes to reach a sport-specific body composition, but during the competition phase, body composition should not be modified anymore since it is typically already at its optimum. There was one study in which dietary intake was strictly controlled since the subjects were in confinement. Brouns et al. simulated a Tour de France race in a metabolic chamber and calculated the daily energy balance from the energy expended and energy intake as calculated from daily food and fluid consumption [73]. They found a positive energy balance during active rest days whereas during the exercise days, a significant negative energy balance was observed. The authors concluded that if prolonged intensive cycling increases energy expenditure to levels above a certain threshold (probably around 20 MJ or 4780 kcal), athletes are unable to consume enough conventional food to provide adequate energy to compensate for the increased energy expenditure. The authors of a recent review addressing the criticisms regarding the value of self-reported dietary intake data reasoned that these should not be used as a measure of energy intake [74]. Our analysis supports this statement since, for athletes, relative energy deficits amounted up to 48% of TEE in female athletes and 33% in male athletes during the competition phase. Thus, there is an urgent need for better methods of dietary intake quantification, such as dietary biomarkers and automated image analysis of food and drink consumption [74]. The classical concept of energy balance, defined as dietary energy intake minus TEE, has been criticized, since according to this definition energy balance is the amount of dietary energy added to or lost from the body’s energy stores after the body’s physiological systems have done their work for the day [75]. Thus, energy balance is an *output* from those systems. In contrast, energy availability, defined as the dietary energy intake minus the energy expended during exercise, is an *input* to the body’s physiological systems, since energy availability is the amount of dietary energy remaining for all other metabolic processes [75]. Endurance athletes, especially female athletes, show low energy availability (<30 kcal/kg FFM/day) [76] and increased risk for changes of the endocrine system affecting energy and bone metabolism, as well as in the cardiovascular and reproductive systems [77]. In healthy young adults, energy balance = 0 kcal/day when energy availability = 45 kcal/kg FFM/day [75]. Since the results of the present study indicate a high negative energy balance in endurance athletes, we must assume that the athletes also demonstrate low energy availability. However, due to the limited data, it was not possible to account for other clinical markers (e.g., bone mineral density), menstrual status, or prevalence of eating disorders in the athletes. We recommend that energy balance-related studies in endurance athletes should also assess and report clinical markers, such as bone mineral density and menstrual status, in order to assess the clinical consequences of the mismatch of TEE and energy intake.

The aggregate analysis yielded a surprising finding. In male endurance athletes, the absolute and relative fat mass was highest during the competition phase. In contrast, during the transition phase, FFM was lowest, which goes along with our expectations with a decrease in exercise volume and intensity. For the female athletes, we did not find these fluctuations in body composition, except for a higher body fat content during the preparation phase compared to the transition phase. We believe that these findings are due to the paucity of data and to the fact that the number and type of athletes varied between seasonal training phases. Indeed, when separately analyzing the few studies where body mass and composition were assessed during both the preparation and competition phases (*N* = 5), both male and female endurance athletes showed a significantly lower percentage of body fat and higher FFM during the competition phase. Further studies with longitudinal assessments of body composition are required to support these findings. However, in only 5.7% of the studies, where body composition was assessed, satisfactory details about standardization were provided. According to Nana et al., studies involving DXA scans of body composition should report details of the DXA machine and software, subject presentation and positioning protocols, and analysis protocols [30]. It has been shown that the use of a non-standardized protocol increased the variability for total and fat-free soft tissue mass compared to a standard protocol, which might include a loss in ability to detect an effect of an intervention that might have relevance for sports performance [78]. The use of non-standardized protocols and the concomitant higher variability might explain some of the unexpected findings of body composition changes in athletes of the present study.

In male endurance athletes, absolute energy intake was higher during the competition phase compared to the preparation phase. The relative energy intake was not different, which can be explained by the apparent significant increase of body mass during the competition phase, and is likely an artifact of the aggregation of data from various studies. In female athletes, neither absolute nor relative energy intake was different between seasonal phases. When focusing on longitudinal studies that assessed energy intake during different training seasons in the same cohort, there was a tendency for male athletes to show greater fluctuations in energy intake. In female cross-country skiers, the energy intake was higher during the preparation phase [50], whereas in female runners and swimmers, the energy intake was higher during the competition phase [47]. However, summing up both studies, no significant differences between training season phases were found. In contrast, male endurance athletes showed a significantly higher energy intake during the competition phase, as seen in male runners [44], cross-country skiers [50], swimmers [43], and triathletes [49]. Although some of the included studies showed greater energy intake in male endurance athletes during the preparation phase (cyclists [46, 48], swimmers [43]), the power of these studies was too low to change the results. However, since energy intake varies in male endurance athletes depending on the training season phase, it indeed seems appropriate to adapt dietary recommendations according to the different training season phases, as proposed by Stellingwerff et al. [17, 18].

### Strengths and Limitations

This is, to our knowledge, the first systematic review focusing on fluctuations in TEE, energy intake, and body composition in endurance athletes. To increase the robustness of the outcomes of our systematic review, we excluded articles where body composition was estimated by skinfold measurements and equations. The accuracy of skinfold measurements depends on the number of measurement sites and the formula used to calculate the percentage of body fat [33]. Since there are many different techniques [79], it is impossible to compare results accurately between studies. Furthermore, skinfold measurements cannot be used to assess intra-abdominal adipose tissue and are highly variable when assessors with limited training and experience perform the measurements [32]. Of course, since skinfolds are very often used for body composition assessments, the exclusion of these articles reduced the total number of articles measuring body composition, which were included in the present systematic review. The inclusion of articles with skinfold body composition determination would have led to a higher number of study estimates and comparisons of different seasonal training phases would have a higher explanatory power. The same is true for estimations of TEE. We included only articles measuring TEE in a more objective way (such as DLW) and excluded articles where TEE was assessed by questionnaires or activity records. This led to the inclusion of a limited number of high-quality studies.

Limitations of the present study relate to the limited cumulative number of subjects, which provided a low explanatory power, and the classification of the different seasonal training phases. In the literature, several similar-sounding terms have been used to describe time points of data collection in athletes. However, assigning the appropriate classification into one of the three seasonal training phases is essential and has a great impact on the final analysis. Furthermore, if articles reported several time points of data collection within one seasonal training phase, we included only the first time point into the analysis in order to assure standardization and avoid selection bias. The exclusion of other time points might have led to the loss of interesting data.

## Conclusions

Our analysis highlights the important seasonal fluctuations in TEE, energy intake, and body composition in male and female endurance athletes across the training season. Therefore, dietary intake recommendations should take into consideration other factors including the actual training load, TEE, and body composition goals of the athlete. The present review supports the statement of the current position stand of the American College of Sports Medicine (ACSM) that energy and nutrient requirements are not static and that periodized dietary recommendations should be developed [9]. Importantly, our analysis again shows the uselessness of self-reported dietary intake, a well-known limitation to energy balance studies, in endurance athletes. The important underreporting suggested by our analysis again raises the question of whether self-reported energy intake data should be used for the determination of energy intake and illustrates the need for more valid and applicable energy intake assessment methods in free-living humans [74]. Since we observed a lack of data during the transition phase, future research should focus on the assessment of TEE, energy intake, and body composition on a reduction in training intensity and volume, such as at the end of the competitive season. In addition, future studies dealing with energy balance and nutrient intake in elite endurance athletes should always mention the time point of data assessments (e.g., seasonal training phase).

## Additional files

Additional file 1: Search strategies in SPORTDiscus and MEDLINE. (PDF 140 kb)
Additional file 2: Results of methodological quality assessment undertaken on included studies. (PDF 276 kb)
Additional file 3: List of excluded references and reason for exclusion. (PDF 490 kb)

## References

[1] Ravussin E, Bogardus C (1989). Relationship of genetics, age, and physical fitness to daily energy expenditure and fuel utilization. Am J Clin Nutr.

[2] Westerterp KR. Physical activity and physical activity induced energy expenditure in humans: measurement, determinants, and effects. Front Physiol. 2013;4:90. 10.3389/fphys.2013.00090 PMC3636460 23637685

[3] Billat VL, Demarle A, Slawinski J, Paiva M, Koralsztein JP (2001). Physical and training characteristics of top-class marathon runners. Med Sci Sports Exerc.

[4] Stellingwerf T (2012). Case study: Nutrition and training periodization in three elite marathon runners. Int J Sport Nutr Exerc Metab.

[5] Zapico AG, Calderon FJ, Benito PJ, Gonzalez CB, Parisi A, Pigozzi F (2007). Evolution of physiological and haematological parameters with training load in elite male road cyclists: a longitudinal study. J Sports Med Phys Fitness.

[6] Fiskerstrand A, Seiler KS (2004). Training and performance characteristics among Norwegian international rowers 1970-2001. Scand J Med Sci Sports.

[7] Neal CM, Hunter AM, Galloway SD (2011). A 6-month analysis of training-intensity distribution and physiological adaptation in Ironman triathletes. J Sports Sci.

[8] Westerterp KR, Saris WH, van Es M, ten Hoor F (1986). Use of the doubly labeled water technique in humans during heavy sustained exercise. J Appl Physiol (1985).

[9] Thomas DT, Erdman KA, Burke LM (2016). American College of Sports Medicine Joint Position Statement. Nutrition and Athletic Performance. Med Sci Sports Exerc.

[10] O'Connor H, Slater G, Lanham-New S, Stear S, Sherriffs M, Collins A (2011). Losing, gaining and making weight for athletes. Sport and exercise nutrition.

[11] Fudge BW, Westerterp KR, Kiplamai FK, Onywera VO, Boit MK, Kayser B (2006). Evidence of negative energy balance using doubly labelled water in elite Kenyan endurance runners prior to competition. Br J Nutr.

[12] Sundgot-Borgen J, Meyer NL, Lohman TG, Ackland TR, Maughan RJ, Stewart AD (2013). How to minimise the health risks to athletes who compete in weight-sensitive sports review and position statement on behalf of the Ad Hoc Research Working Group on Body Composition, Health and Performance, under the auspices of the IOC Medical Commission. Br J Sports Med.

[13] World Health Organization (WHO) (2000). Obesity: preventing and managing the global epidemic. Report of a WHO Consultation.

[14] Issurin VB (2010). New horizons for the methodology and physiology of training periodization. Sports Med.

[15] Matveyev L (1975). Periodisierung des sportlichen Trainings.

[16] Bompa T, Haff G (2009). Periodization. Theory and methodology of training. 5th ed.

[17] Stellingwerff T, Boit MK, Res PT (2007). Nutritional strategies to optimize training and racing in middle-distance athletes. J Sports Sci.

[18] Stellingwerff T, Maughan RJ, Burke LM (2011). Nutrition for power sports: middle-distance running, track cycling, rowing, canoeing/kayaking, and swimming. J Sports Sci.

[19] Burke LM, Hawley JA, Wong SH, Jeukendrup AE (2011). Carbohydrates for training and competition. J Sports Sci.

[20] Maughan RJ, Burke LM (2011). Practical nutritional recommendations for the athlete. Nestle Nutr Inst Workshop Ser.

[21] Rodriguez NR, Di Marco NM, Langley S (2009). American College of Sports Medicine position stand. Nutrition and athletic performance. Med Sci Sports Exerc.

[22] Burke LM, Mujika I (2014). Nutrition for recovery in aquatic sports. Int J Sport Nutr Exerc Metab.

[23] Mujika I, Stellingwerff T, Tipton K (2014). Nutrition and training adaptations in aquatic sports. Int J Sport Nutr Exerc Metab.

[24] Shaw G, Koivisto A, Gerrard D, Burke LM (2014). Nutrition considerations for open-water swimming. Int J Sport Nutr Exerc Metab.

[25] Shaw G, Boyd KT, Burke LM, Koivisto A (2014). Nutrition for swimming. Int J Sport Nutr Exerc Metab.

[26] Burke LM, Millet G, Tarnopolsky MA (2007). Nutrition for distance events. J Sports Sci.

[27] Jeukendrup AE (2011). Nutrition for endurance sports: marathon, triathlon, and road cycling. J Sports Sci.

[28] Vilaca KH, Ferriolli E, Lima NK, Paula FJ, Moriguti JC (2009). Effect of fluid and food intake on the body composition evaluation of elderly persons. J Nutr Health Aging.

[29] Lohman M, Tallroth K, Kettunen JA, Marttinen MT (2009). Reproducibility of dual-energy x-ray absorptiometry total and regional body composition measurements using different scanning positions and definitions of regions. Metabolism.

[30] Nana A, Slater GJ, Stewart AD, Burke LM (2015). Methodology review: using dual-energy X-ray absorptiometry (DXA) for the assessment of body composition in athletes and active people. Int J Sport Nutr Exerc Metab.

[31] Saunders MJ, Blevins JE, Broeder CE (1998). Effects of hydration changes on bioelectrical impedance in endurance trained individuals. Med Sci Sports Exerc.

[32] Madden AM, Smith S (2016). Body composition and morphological assessment of nutritional status in adults: a review of anthropometric variables. J. Hum. Nutr. Diet..

[33] Temple D, Denis R, Walsh MC, Dicker P, Byrne AT (2015). Comparison of anthropometric-based equations for estimation of body fat percentage in a normal-weight and overweight female cohort: validation via air-displacement plethysmography. Public Health Nutr.

[34] Magkos F, Yannakoulia M (2003). Methodology of dietary assessment in athletes: concepts and pitfalls. Curr Opin Clin Nutr Metab Care.

[35] Bemben DA, Buchanan TD, Bemben MG, Knehans AW (2004). Influence of type of mechanical loading, menstrual status, and training season on bone density in young women athletes. J Strength Cond Res.

[36] Carbuhn AF, Fernandez TE, Bragg AF, Green JS, Crouse SF (2010). Sport and training influence bone and body composition in women collegiate athletes. J Strength Cond Res.

[37] Kabasakalis A, Kalitsis K, Tsalis G, Mougios V (2007). Imbalanced nutrition of top-level swimmers. Int J Sports Med.

[38] LaForgia J, Withers RT, Williams AD, Murch BJ, Chatterton BE, Schultz CG (1999). Effect of 3 weeks of detraining on the resting metabolic rate and body composition of trained males. Eur J Clin Nutr.

[39] Loftin M, Warren B, Mayhew J (1992). Comparison of physiologic and performance variables in male and female cross-country runners during a competitive season. Sports Med Train Rehabil.

[40] Noland RC, Baker JT, Boudreau SR, Kobe RW, Tanner CJ, Hickner RC (2001). Effect of intense training on plasma leptin in male and female swimmers. Med Sci Sports Exerc.

[41] Siders WA, Bolonchuk WW, Lukaski HC (1991). Effects of participation in a collegiate sport season on body composition. J Sports Med Phys Fitness.

[42] Siders WA, Lukaski HC, Bolonchuk WW (1993). Relationships among swimming performance, body composition and somatotype in competitive collegiate swimmers. J Sports Med Phys Fitness.

[43] Barr SI, Costill DL (1992). Effect of increased training volume on nutrient intake of male collegiate swimmers. Int J Sports Med.

[44] Couzy F, Lafargue P, Guezennec CY (1990). Zinc metabolism in the athlete: influence of training, nutrition and other factors. Int J Sports Med.

[45] Desgorces FD, Chennaoui M, Gomez-Merino D, Drogou C, Guezennec CY (2004). Leptin response to acute prolonged exercise after training in rowers. Eur J Appl Physiol.

[46] Garcia-Roves PM, Terrados N, Fernandez S, Patterson AM (2000). Comparison of dietary intake and eating behavior of professional road cyclists during training and competition. Int J Sport Nutr Exerc Metab.

[47] Hassapidou MN, Manstrantoni A (2001). Dietary intakes of elite female athletes in Greece. J Hum Nutr Dietetics.

[48] Jensen CD, Zaltas ES, Whittam JH (1992). Dietary intakes of male endurance cyclists during training and racing. J Am Diet Assoc.

[49] Margaritis I, Palazzetti S, Rousseau AS, Richard MJ, Favier A (2003). Antioxidant supplementation and tapering exercise improve exercise-induced antioxidant response. J Am Coll Nutr.

[50] Papadopoulou SK, Gouvianaki A, Grammatikopoulou MG, Maraki Z, Pagkalos IG, Malliaropoulos N (2012). Body composition and dietary intake of elite cross-country skiers members of the greek national team. Asian J Sports Med.

[51] Peters EM, Goetzsche JM (1997). Dietary practices of South African ultradistance runners. Int J Sport Nutr.

[52] Taylor SR, Rogers GG, Driver HS (1997). Effects of training volume on sleep, psychological, and selected physiological profiles of elite female swimmers. Med Sci Sports Exerc.

[53] Stroup DF, Berlin JA, Morton SC, Olkin I, Williamson GD, Rennie D (2000). Meta-analysis of observational studies in epidemiology: a proposal for reporting. Meta-analysis Of Observational Studies in Epidemiology (MOOSE) group. JAMA.

[54] Orwin R, Cooper H, Hedges L (1994). Evaluating coding decisions. The handbook of research synthesis.

[55] Downs SH, Black N (1998). The feasibility of creating a checklist for the assessment of the methodological quality both of randomised and non-randomised studies of health care interventions. J Epidemiol Community Health.

[56] Fox AS, Bonacci J, McLean SG, Spittle M, Saunders N (2014). What is normal? Female lower limb kinematic profiles during athletic tasks used to examine anterior cruciate ligament injury risk: a systematic review. Sports Med.

[57] Wang ZM, Pierson RN, Heymsfield SB (1992). The five-level model: a new approach to organizing body-composition research. Am J Clin Nutr.

[58] Higgins, Green, editors. Cochrane Handbook for Systematic Reviews of Interventions. Chichester, West Sussex, England: Wiley-Blackwell 2012

[59] Gravetter F, Wallnau L (2013). Essentials of statistics for the behavioral sciences.

[60] Bescós R, Rodríguez FA, Iglesias X, Knechtle B, Benítez A, Marina M (2012). Nutritional behavior of cyclists during a 24-hour team relay race: a field study report. Journal of the International Society of Sports Nutrition.

[61] Rehrer NJ, Hellemans IJ, Rolleston AK, Rush E, Miller BF (2010). Energy intake and expenditure during a 6-day cycling stage race. Scand J Med Sci Sports.

[62] Hulton AT, Lahart I, Williams KL, Godfrey R, Charlesworth S, Wilson M (2010). Energy expenditure in the Race Across America (RAAM). Int J Sports Med.

[63] Costa RJ, Gill SK, Hankey J, Wright A, Marczak S (2014). Perturbed energy balance and hydration status in ultra-endurance runners during a 24 h ultra-marathon. Br J Nutr.

[64] Morris FL, Payne WR (1996). Seasonal variations in the body composition of lightweight rowers. Br J Sports Med.

[65] Ormsbee MJ, Arciero PJ (2012). Detraining increases body fat and weight and decreases VO2peak and metabolic rate. J Strength Cond Res.

[66] Boulay MR, Serresse O, Almeras N, Tremblay A (1994). Energy expenditure measurement in male cross-country skiers: comparison of two field methods. Med Sci Sports Exerc.

[67] Sjodin AM, Andersson AB, Hogberg JM, Westerterp KR (1994). Energy balance in cross-country skiers: a study using doubly labeled water. Med Sci Sports Exerc.

[68] Schulz LO, Alger S, Harper I, Wilmore JH, Ravussin E (1992). Energy expenditure of elite female runners measured by respiratory chamber and doubly labeled water. J Appl Physiol.

[69] Hill RJ, Davies PS (2002). Energy intake and energy expenditure in elite lightweight female rowers. Med Sci Sports Exerc.

[70] Trappe TA, Gastaldelli A, Jozsi AC, Troup JP, Wolfe RR (1997). Energy expenditure of swimmers during high volume training. Med Sci Sports Exerc.

[71] Winters KM, Adams WC, Meredith CN, Loan MD, Lasley BL (1996). Bone density and cyclic ovarian function in trained runners and active controls. Med Sci Sports Exerc.

[72] Thompson FE, Byers T (1994). Dietary assessment resource manual. J Nutr.

[73] Brouns F, Saris WH, Stroecken J, Beckers E, Thijssen R, Rehrer NJ (1989). Eating, drinking, and cycling. A controlled Tour de France simulation study, Part I. Int J Sports Med.

[74] Subar AF, Freedman LS, Tooze JA, Kirkpatrick SI, Boushey C, Neuhouser ML (2015). Addressing current criticism regarding the value of self-report dietary data. J Nutr.

[75] Loucks AB, Kiens B, Wright HH (2011). Energy availability in athletes. J Sports Sci.

[76] Loucks AB (2007). Low energy availability in the marathon and other endurance sports. Sports Med.

[77] Melin A, Tornberg AB, Skouby S, Moller SS, Sundgot-Borgen J, Faber J (2015). Energy availability and the female athlete triad in elite endurance athletes. Scand J Med Sci Sports.

[78] Nana A, Slater GJ, Hopkins WG, Halson SL, Martin DT, West NP (2016). Importance of standardized DXA protocol for assessing physique changes in athletes. Int J Sport Nutr Exerc Metab.

[79] Ball SD, Altena TS, Swan PD (2004). Comparison of anthropometry to DXA: a new prediction equation for men. Eur J Clin Nutr.

[80] Armstrong LE, Casa DJ, Emmanuel H, Ganio MS, Klau JF, Lee EC (2012). Nutritional, physiological, and perceptual responses during a summer ultraendurance cycling event. J Strength Cond Res.

[81] Berg U, Enqvist JK, Mattsson CM, Carlsson-Skwirut C, Sundberg CJ, Ekblom B (2008). Lack of sex differences in the IGF-IGFBP response to ultra endurance exercise. Scand J Med Sci Sports.

[82] Brewer CP, Dawson B, Wallman KE, Guelfi KJ (2013). Effect of repeated sodium phosphate loading on cycling time-trial performance and VO2peak. Int J Sport Nutr Exerc Metab.

[83] Brinkworth GD, Buckley JD, Bourdon PC, Gulbin JP, David A (2002). Oral bovine colostrum supplementation enhances buffer capacity but not rowing performance in elite female rowers. Int J Sport Nutr Exerc Metab.

[84] Decombaz J, Gmuender B, Sierro G, Cerretelli P (1992). Muscle carnitine after strenuous endurance exercise. J Appl Physiol.

[85] Dellavalle DM, Haas JD (2014). Iron supplementation improves energetic efficiency in iron-depleted female rowers. Med Sci Sports Exerc.

[86] Desgorces FD, Chennaoui M, Drogou C, Guezennec CY, Gomez-Merino D (2008). Relationships between leptin levels and carbohydrate intake during rowing training. J Sports Med Phys Fitness.

[87] Drenowatz C, Eisenmann JC, Carlson JJ, Pfeiffer KA, Pivarnik JM (2012). Energy expenditure and dietary intake during high-volume and low-volume training periods among male endurance athletes. Appl Physiol Nutr Metab.

[88] Drenowatz C, Eisenmann JC, Pivarnik JM, Pfeiffer KA, Carlson JJ (2013). Differences in energy expenditure between high- and low-volume training. Eur J Sport Sci.

[89] Emhoff CA, Messonnier LA, Horning MA, Fattor JA, Carlson TJ, Brooks GA (2013). Gluconeogenesis and hepatic glycogenolysis during exercise at the lactate threshold. J Appl Physiol.

[90] Enqvist JK, Mattsson CM, Johansson PH, Brink-Elfegoun T, Bakkman L, Ekblom BT (2010). Energy turnover during 24 hours and 6 days of adventure racing. J Sports Sci.

[91] Fudge BW, Easton C, Kingsmore D, Kiplamai FK, Onywera VO, Westerterp KR (2008). Elite Kenyan endurance runners are hydrated day-to-day with ad libitum fluid intake. Med Sci Sports Exerc.

[92] Garcia-Roves PM, Terrados N, Fernandez SF, Patterson AM (1998). Macronutrients intake of top level cyclists during continuous competition--change in the feeding pattern. Int J Sports Med.

[93] Gorsuch J, Long J, Miller K, Primeau K, Rutledge S, Sossong A (2013). The effect of squat depth on multiarticular muscle activation in collegiate cross-country runners. J Strength Cond Res.

[94] Griffith RO, Dressendorfer RH, Fullbright GD, Wade CE (1990). Testicular function during exhaustive endurance training. / La fonction testiculaire lors d ' un entrainement epuisant d ' endurance. Phys Sportsmed.

[95] Havemann L, Goedecke JH (2008). Nutritional practices of male cyclists before and during an ultraendurance event. Int J Sport Nutr Exerc Metab.

[96] Heinonen A, Oja P, Kannus P, Sievanen H, Manttari A, Vuori I (1993). Bone mineral density of female athletes in different sports. Bone Miner.

[97] Herring JL, Mole PA, Meredith CN, Stern JS (1992). Effect of suspending exercise training on resting metabolic rate in women. Med Sci Sports Exerc.

[98] Jones PJ, Leitch CA (1993). Validation of doubly labeled water for measurement of caloric expenditure in collegiate swimmers. J Appl Physiol.

[99] Jurimae J, Jurimae T, Pihl E (1999). Rowing ergometer performance and anaerobic capacity in college rowers. Kinesiology.

[100] Jurimae J, Hofmann P, Jurimae T, Maestu J, Purge P, Wonisch M (2006). Plasma adiponectin response to sculling exercise at individual anaerobic threshold in college level male rowers. Int J Sports Med.

[101] Jurimae J, Jurimae T (2004). Plasma leptin responses to prolonged sculling in female rowers. J Sports Med Phys Fitness.

[102] Jurimae J, Purge P, Jurimae T (2007). Effect of prolonged training period on plasma adiponectin in elite male rowers. Horm Metab Res.

[103] Jurimae J, Ramson R, Maestu J, Jurimae T, Arciero PJ, Braun WA (2011). Interactions between adipose, bone, and muscle tissue markers during acute negative energy balance in male rowers. J Sports Med Phys Fitness.

[104] Koshimizu T, Matsushima Y, Yokota Y, Yanagisawa K, Nagai S, Okamura K (2012). Basal metabolic rate and body composition of elite Japanese male athletes. J Med Invest.

[105] Lazzer S, Salvadego D, Rejc E, Buglione A, Antonutto G, di Prampero PE (2012). The energetics of ultra-endurance running. Eur J Appl Physiol.

[106] Maestu J, Jurimae J, Purge P, Ramson R, Jurimae T (2010). Performance improvement is associated with higher postexercise responses in interleukin-6 and tumor necrosis factor concentrations. J Sports Med Phys Fitness.

[107] Magkos F, Yannakoulia M, Kavouras SA, Sidossis LS (2007). The type and intensity of exercise have independent and additive effects on bone mineral density. Int J Sports Med.

[108] Maïmoun L, Manetta P, Leroux S (2003). Testosterone is significantly reduced in endurance athletes without impact on bone mineral density. Horm Res.

[109] Martin MK, Martin DT, Collier GR, Burke LM (2002). Voluntary food intake by elite female cyclists during training and racing: influence of daily energy expenditure and body composition. Int J Sport Nutr Exerc Metab.

[110] Medelli J, Lounana J, Menuet JJ, Shabani M, Cordero-MacIntyre Z (2009). Is osteopenia a health risk in professional cyclists?. J Clin Densitom.

[111] Moses K, Manore MM (1991). Development and testing of a carbohydrate monitoring tool for athletes. J Am Diet Assoc.

[112] Motonaga K, Yoshida S, Yamagami F, Kawano T, Takeda E (2006). Estimation of total daily energy expenditure and its components by monitoring the heart rate of Japanese endurance athletes. J Nutr Sci Vitaminol (Tokyo).

[113] Muoio DM, Leddy JJ, Horvath PJ, Awad AB, Pendergast DR (1994). Effect of dietary fat on metabolic adjustments to maximal VO2 and endurance in runners. Med Sci Sports Exerc.

[114] Ousley-Pahnke L, Black DR, Gretebeck RJ (2001). Dietary intake and energy expenditure of female collegiate swimmers during decreased training prior to competition. J Am Diet Assoc.

[115] Palazzetti S, Rousseau AS, Richard MJ, Favier A, Margaritis I (2004). Antioxidant supplementation preserves antioxidant response in physical training and low antioxidant intake. Br J Nutr.

[116] Palm R, Jürimäe J, Mästu J, Purge P, Jürimäe T, Rom K (2005). Relationship between body composition and aerobic capacity values in well-trained male rowers. Acta Kinesiol Universitatis Tartu.

[117] Penteado VS, Castro CH, Pinheiro Mde M, Santana M, Bertolino S, de Mello MT (2010). Diet, body composition, and bone mass in well-trained cyclists. J Clin Densitom.

[118] Phillips SM, Atkinson SA, Tarnopolsky MA, MacDougall JD (1993). Gender differences in leucine kinetics and nitrogen balance in endurance athletes. J Appl Physiol (1985).

[119] Roberts D, Smith DJ (1992). Training at moderate altitude: iron status of elite male swimmers. J Lab Clin Med.

[120] Santos DA, Dawson JA, Matias CN, Rocha PM, Minderico CS, Allison DB (2014). Reference values for body composition and anthropometric measurements in athletes. PLoS One.

[121] Sato A, Shimoyama Y, Ishikawa T, Murayama N (2011). Dietary thiamin and riboflavin intake and blood thiamin and riboflavin concentrations in college swimmers undergoing intensive training. Int J Sport Nutr Exerc Metab.

[122] Schena F, Pattini A, Mantovanelli S, Kies CV, Driskell JA (1995). Iron status in athletes involved in endurance and in prevalently anaerobic sports. Sports nutrition: minerals and electrolytes.

[123] Schenk K, Gatterer H, Ferrari M, Ferrari P, Cascio VL, Burtscher M (2010). Bike Transalp 2008: liquid intake and its effect on the body's fluid homeostasis in the course of a multistage, cross-country, MTB marathon race in the central Alps. Clin J Sport Med.

[124] Sherman WM, Doyle JA, Lamb DR, Strauss RH (1993). Dietary carbohydrate, muscle glycogen, and exercise performance during 7 d of training. Am J Clin Nutr.

[125] Simsch C, Lormes W, Petersen KG, Baur S, Liu Y, Hackney AC (2002). Training intensity influences leptin and thyroid hormones in highly trained rowers. Int J Sports Med.

[126] Sundby OH, Gorelick ML S (2014). Relationship between functional hamstring: quadriceps ratios and running economy in highly trained and recreational female runners. J Strength Cond Res.

[127] Tomten SE, Hostmark AT (2006). Energy balance in weight stable athletes with and without menstrual disorders. Scand J Med Sci Sports.

[128] Vaiksaar S, Jurimae J, Maestu J, Purge P, Kalytka S, Shakhlina L (2011). No effect of menstrual cycle phase on fuel oxidation during exercise in rowers. Eur J Appl Physiol.

[129] Witard OC, Jackman SR, Kies AK, Jeukendrup AE, Tipton KD (2011). Effect of increased dietary protein on tolerance to intensified training. Med Sci Sports Exerc.

[130] Yeater R, Reed C, Ullrich I, Morise A, Borsch M (1996). Resistance trained athletes using or not using anabolic steroids compared to runners: effects on cardiorespiratory variables, body composition, and plasma lipids. Br J Sports Med.

[131] Zajac A, Poprzecki S, Maszczyk A, Czuba M, Michalczyk M, Zydek G (2014). The effects of a ketogenic diet on exercise metabolism and physical performance in off-road cyclists. Nutrients.

[132] Zalcman I, Guarita HV, Juzwiak CR, Crispim CA, Antunes HK, Edwards B (2007). Nutritional status of adventure racers. Nutrition.

