# Supplementary material for: Total Energy Expenditure, Energy Intake, and Body Composition in Endurance Athletes Across the Training Season: A Systematic Review
Source: Sports Med Open. 2017 Feb 4;3:8. doi: 10.1186/s40798-017-0076-1 (PMC5292109; doi:10.1186/s40798-017-0076-1)
Supplement: Additional file 1: — Search strategies in SPORTDiscus and MEDLINE. (PDF 140 kb) [file 40798_2017_76_MOESM1_ESM.pdf]

Article title: Total Energy Expenditure, Energy Intake and Body Composition in Endurance Athletes across the Training Season: A Systematic Review.

Authors: Juliane Heydenreich, Bengt Kayser, Yves Schutz, Katarina Melzer

Journal name: Sports Medicine

Corresponding author: Juliane Heydenreich, Swiss Federal Institute of Sport, Hauptstrasse 247, 2532 Magglingen, Switzerland and University of Lausanne, Faculty of Biology and Medicine, Rue du Bugnon 21, 1011 Lausanne, Switzerland, e-mail: juliane.heydenreich@googlemail.com

Online Resource 1 – Search strategies in SPORTDiscus and MEDLINE

SPORTDiscus with Full Text via EBSCOHost (Date of search: January 31st, 2015)

#	Query / Limiters / Expanders
S1	endurance athletes
S2	endurance-trained
S3	endurance trained
S4	aerobically trained
S5	runners
S6	swimmers
S7	triathletes
S8	skiers
S9	cyclists
S10	rowers
S11	S1 OR S2 OR S3 OR S4 OR S5 OR S6 OR S7 OR S8 OR S9 OR S10
S12	body composition
S13	fat mass
S14	fat-mass
S15	fat free mass
S16	fat-free mass
S17	body fat
S18	metabolic rate
S19	energy expenditure
S20	dietary intake
S21	food intake
S22	energy intake
S23	food consumption
S24	macronutrient*
S25	S12 OR S13 OR S14 OR S15 OR S16 OR S17 OR S18 OR S19 OR S20 OR S21 OR S22 OR S23 OR S24
S26	S11 AND S25
	Limiters – Published Date: 19900101-20303112; Peer Reviewed; Language: English
	Search Mode – Boolean/Phrase

MEDLINE via Pubmed (Date of search: January 31st, 2015)

Search ((((((((endurance athletes OR endurance-trained OR endurance trained OR aerobically trained OR runners OR swimmers OR triathletes OR skiers OR cyclists OR rowers)) AND (body composition OR fat mass OR fat-mass OR fat free mass OR fat-free mass OR body fat OR metabolic rate OR energy expenditure OR dietary intake OR food intake OR energy intake OR food consumption OR macronutrient*))) AND ("1990"[Date - Publication] : "2015/01/31"[Date - Publication])) AND Humans[Mesh]))) AND english[Language])
