# Supplementary material for: Total Energy Expenditure, Energy Intake, and Body Composition in Endurance Athletes Across the Training Season: A Systematic Review
Source: Sports Med Open. 2017 Feb 4;3:8. doi: 10.1186/s40798-017-0076-1 (PMC5292109; doi:10.1186/s40798-017-0076-1)
Supplement: Additional file 2: — Results of methodological quality assessment undertaken on included studies. (PDF 276 kb) [file 40798_2017_76_MOESM2_ESM.pdf]

Article title: Total Energy Expenditure, Energy Intake and Body Composition in Endurance Athletes across the Training Season: A Systematic Review.

Authors: Juliane Heydenreich, Bengt Kayser, Yves Schutz, Katarina Melzer

Journal name: Sports Medicine

Corresponding author: Juliane Heydenreich, Swiss Federal Institute of Sport, Hauptstrasse 247, 2532 Magglingen, Switzerland and University of Lausanne, Faculty of Biology and Medicine, Rue du Bugnon 21, 1011 Lausanne, Switzerland, e-mail: juliane.heydenreich@googlemail.com

Online Resource 2 – Results of methodological quality assessment undertaken on included studies.

Reference	1*	2	3	6	7	11	12	16	18	20	Total (/10)
Armstrong et al. 2012 [1]	Y	Y	Y	Y	Y	U	N	Y	Y	Y	8
Barr & Costill 1992 [2]	Y	Y	Y	Y	Y	U	N	Y	Y	Y	8
Bemben et al. 2004 [3]	Y	Y	Y	Y	Y	U	N	Y	Y	Y	8
Berg et al. 2008 [4]	Y	Y	Y	Y	Y	U	N	Y	Y	Y	8
Bescós et al, 2012 [5]	Y	Y	Y	Y	Y	U	N	Y	Y	Y	8
Boulay et al. 1994 [6]	Y	Y	Y	Y	Y	U	N	Y	Y	Y	8
Brewer et al. 2013 [7]	Y	Y	Y	Y	Y	U	N	Y	Y	Y	8
Brinkworth et al. 2002 [8]	Y	N	Y	Y	Y	U	N	Y	Y	Y	7
Carbuhn et al. 2010 [9]	Y	Y	Y	Y	Y	Y	N	Y	Y	Y	9
Costa et al. 2014 [10]	Y	Y	Y	Y	Y	U	N	Y	Y	Y	8
Couzy et al. 1990 [11]	Y	Y	Y	Y	Y	U	N	Y	Y	Y	8
Decombaz et al. 1992 [12]	Y	Y	Y	Y	Y	U	N	Y	Y	Y	8
Dellavalle & Haas 2014 [13]	Y	Y	Y	Y	Y	U	N	Y	Y	Y	8
Desgorces et al. 2004 [14]	Y	Y	N	Y	Y	U	N	Y	Y	Y	7
Desgorces et al. 2008 [15]	Y	Y	N	Y	Y	U	N	Y	Y	Y	7
Drenowatz et al. 2012 [16]	Y	Y	Y	Y	Y	U	N	Y	Y	Y	8
Drenowatz et al. 2013 [17]	Y	Y	Y	Y	Y	U	N	Y	Y	Y	8
Emhoff et al. 2013 [18]	Y	Y	Y	Y	Y	U	N	Y	Y	Y	8

Enqvist et al. 2010 [19]	Y	Y	Y	Y	Y	U	N	Y	Y	Y	8
Fudge et al. 2006 [20]	Y	Y	Y	Y	Y	U	N	Y	Y	Y	8
Fudge et al. 2008 [21]	Y	Y	Y	Y	Y	U	N	Y	Y	Y	8
Garcia-Roves et al. 1998 [22]	Y	Y	Y	Y	Y	U	N	Y	Y	Y	8
Garcia-Roves et al. 2000 [23]	Y	Y	Y	Y	Y	U	N	Y	Y	Y	8
Gorsuch & Long 2013 [24]	Y	Y	Y	Y	Y	U	N	Y	Y	Y	8
Griffith et al. 1990 [25]	Y	Y	Y	Y	Y	U	N	Y	Y	Y	8
Hassapidou & Manstrantoni 2001 [26]	Y	Y	Y	Y	Y	U	N	Y	U	Y	7
Havemann & Goedecke 2008 [27]	Y	Y	Y	Y	Y	U	N	Y	Y	Y	8
Heinonen et al. 1993 [28]	Y	Y	Y	Y	Y	U	N	Y	Y	Y	8
Herring et al. 1992 [29]	Y	Y	Y	Y	Y	U	N	Y	Y	Y	9
Hill & Davies 2002 [30]	Y	Y	Y	Y	Y	U	N	Y	Y	Y	9
Hulton et al. 2010 [31]	Y	Y	Y	Y	Y	U	N	Y	Y	Y	9
Jensen et al. 1992 [32]	Y	Y	N	Y	Y	U	N	Y	Y	Y	8
Jones & Leitch 1993 [33]	Y	Y	Y	Y	Y	U	N	Y	Y	Y	8
Jurimae et al. 1999 [34]	Y	Y	Y	Y	Y	U	N	Y	Y	Y	8
Jurimae et al. 2006 [35]	Y	Y	Y	Y	Y	U	N	Y	Y	Y	8
Jurimae & Jurimae 2004 [36]	Y	Y	Y	Y	Y	U	N	Y	Y	Y	8
Jurimae et al. 2007 [37]	Y	Y	Y	Y	Y	U	N	Y	Y	Y	8
Jurimae et al. 2011 [38]	Y	Y	Y	Y	Y	U	N	Y	Y	Y	8
Kabasakalis et al. 2007 [39]	Y	Y	Y	Y	Y	U	N	Y	Y	Y	8
Koshimizu et al. 2012 [40]	Y	Y	Y	Y	Y	U	N	Y	Y	Y	8
LaForgia et al. 1999 [41]	Y	Y	Y	Y	Y	U	N	Y	Y	Y	8
Lazzer et al. 2012 [42]	Y	Y	Y	Y	Y	U	N	Y	Y	Y	8
Loftin et al. 1992 [43]	Y	Y	Y	Y	Y	U	N	Y	Y	Y	8
Maestu et al. 2010 [44]	Y	Y	Y	Y	Y	U	N	Y	Y	Y	8
Maïmoun et al. 2003 [45]	Y	Y	Y	Y	Y	U	N	Y	Y	Y	8
Magkos et al. 2007 [46]	Y	Y	Y	Y	Y	U	N	Y	Y	Y	8
Margaritis et al. 2003 [47]	Y	Y	Y	Y	Y	U	N	Y	Y	Y	8

Martin et al. 2002 [48]	Y	Y	Y	Y	Y	U	N	Y	Y	Y	8
Medelli et al. 2009 [49]	Y	N	Y	Y	Y	U	N	Y	Y	Y	7
Moses & Manore 1991 [50]	Y	Y	Y	Y	Y	U	N	Y	Y	Y	8
Motonaga et al. 2006 [51]	Y	Y	Y	Y	Y	U	N	Y	Y	Y	8
Muoio et al. 1994 [52]	Y	Y	Y	Y	Y	U	N	Y	Y	Y	8
Noland et al. 2001 [53]	Y	Y	Y	N	Y	U	N	Y	Y	Y	7
Ousley-Pahnke et al. 2001 [54]	N	Y	Y	Y	Y	U	N	Y	Y	Y	7
Palazzetti et al. 2004 [55]	Y	Y	Y	Y	Y	U	N	Y	Y	Y	8
Palm et al. 2005 [56]	Y	Y	Y	Y	Y	U	N	Y	Y	Y	8
Papadopoulou et al. 2012 [57]	Y	Y	Y	Y	Y	U	N	Y	Y	Y	8
Penteado et al. 2010 [58]	Y	Y	Y	Y	Y	Y	N	Y	Y	Y	9
Peters & Goetzsche 1997 [59]	Y	Y	Y	Y	Y	U	N	Y	Y	Y	8
Phillips et al. 1993 [60]	Y	Y	Y	Y	Y	U	N	Y	Y	Y	8
Rehrer et al. 2010 [61]	Y	Y	Y	Y	Y	U	N	Y	Y	Y	8
Roberts & Smith 1992 [62]	Y	Y	Y	Y	Y	U	N	Y	Y	Y	8
Santos et al. 2014 [63]	Y	Y	Y	Y	Y	U	N	Y	Y	Y	8
Sato et al. 2011 [64]	Y	Y	Y	Y	Y	Y	N	Y	Y	Y	9
Schena et al. 1995 [65]	Y	Y	Y	Y	Y	U	N	Y	Y	Y	8
Schenk et al. 2010 [66]	Y	Y	Y	Y	Y	U	N	Y	Y	Y	8
Schulz et al. 1992 [67]	Y	Y	Y	Y	Y	U	N	Y	Y	Y	8
Sherman et al. 1993 [68]	Y	N	Y	Y	Y	U	N	Y	Y	Y	7
Siders et al. 1991 [69]	Y	Y	Y	Y	Y	U	N	Y	Y	Y	8
Siders et al. 1993 [70]	Y	Y	Y	Y	Y	U	N	Y	Y	Y	8
Simsch et al. 2002 [71]	Y	N	Y	Y	Y	U	N	Y	Y	Y	7
Sjodin et al. 1994 [72]	Y	Y	Y	Y	Y	U	N	Y	Y	Y	8
Sundby & Gorelick 2014 [73]	Y	Y	Y	Y	Y	U	N	Y	Y	Y	8
Taylor et al. 1997 [74]	Y	Y	Y	Y	Y	U	N	Y	Y	Y	8
Tomten & Hostmark 2006 [75]	Y	Y	Y	Y	Y	U	N	Y	Y	Y	8
Trappe et al. 1997 [76]	Y	Y	Y	Y	Y	U	N	Y	Y	Y	8

Vaiksaar et al. 2011 [77]	Y	Y	Y	Y	Y	U	N	Y	Y	Y	8
Winters et al. 1996 [78]	Y	Y	Y	Y	Y	U	N	Y	Y	Y	8
Witard et al. 2011 [79]	Y	Y	Y	Y	Y	U	N	Y	Y	Y	8
Yeater et al. 1996 [80]	Y	Y	Y	Y	Y	U	N	Y	Y	Y	8
Zajac et al. 2014 [81]	Y	Y	Y	Y	Y	U	N	Y	Y	Y	8
Zalcman et al. 2007 [82]	Y	Y	Y	Y	Y	U	N	Y	Y	Y	8

*Numbers in this row refer to the question number in the Downs and Black scale [83]. The questions appear below. Y = yes, N = no, U = unable to determine.

1 = Is the hypothesis/aim/objective of the study clearly described?

2 = Are the main outcomes to be measured clearly described in the Introduction or Methods section?

3 = Are the characteristics of the patients included in the study clearly described?

6 = Are the main findings of the study clearly described?

7 = Does the study provide estimates of the random variability in the data for the main outcomes?

11 = Were the subjects asked to participate in the study representative of the entire population from which they were recruited?

12 = Were those subjects who were prepared to participate representative of the entire population from which they were recruited?

16 = If any of the results of the study were based on "data dredging", was this made clear?

18 = Were the statistical tests used to assess the main outcomes appropriate?

20 = Were the main outcome measures used accurate (valid and reliable)?

References

1. Armstrong LE, Casa DJ, Emmanuel H, Ganio MS, Klau JF, Lee EC et al. Nutritional, physiological, and perceptual responses during a summer ultraendurance cycling event. J Strength Cond Res. 2012;26(2):307-18.

2. Barr SI, Costill DL. Effect of increased training volume on nutrient intake of male collegiate swimmers. Int J Sports Med. 1992;13(1):47-51.

3. Bemben DA, Buchanan TD, Bemben MG, Knehans AW. Influence of type of mechanical loading, menstrual status, and training season on bone density in young women athletes. J Strength Cond Res. 2004;18(2):220-6.

4. Berg U, Enqvist JK, Mattsson CM, Carlsson-Skwirut C, Sundberg CJ, Ekblom B et al. Lack of sex differences in the IGF-IGFBP response to ultra endurance exercise. Scand J Med Sci Sports. 2008;18(6):706-14.

5. Bescós R, Rodríguez FA, Iglesias X, Knechtle B, Benítez A, Marina M et al. Nutritional behavior of cyclists during a 24-hour team relay race: a field study report. Journal of the International Society of Sports Nutrition. 2012;9(1):1-11.

6. Boulay MR, Serresse O, Almeras N, Tremblay A. Energy expenditure measurement in male cross-country skiers: comparison of two field methods. Med Sci Sports Exerc. 1994;26(2):248-53.

7. Brewer CP, Dawson B, Wallman KE, Guelfi KJ. Effect of Repeated Sodium Phosphate Loading on Cycling Time-Trial Performance and VO2peak. Int J Sport Nutr Exerc Metab. 2013;23(2):187-94.

8. Brinkworth GD, Buckley JD, Bourdon PC, Gulbin JP, David A. Oral bovine colostrum supplementation enhances buffer capacity but not rowing performance in elite female rowers. Int J Sport Nutr Exerc Metab. 2002;12(3):349-65.

9. Carbuhn AF, Fernandez TE, Bragg AF, Green JS, Crouse SF. Sport and training influence bone and body composition in women collegiate athletes. J Strength Cond Res. 2010;24(7):1710-7.

10. Costa RJ, Gill SK, Hankey J, Wright A, Marczak S. Perturbed energy balance and hydration status in ultra-endurance runners during a 24 h ultra-marathon. Br J Nutr. 2014;112(3):428-37.

11. Couzy F, Lafargue P, Guezennec CY. Zinc metabolism in the athlete: influence of training, nutrition and other factors. Int J Sports Med. 1990;11(4):263-6.

12. Decombaz J, Gmuender B, Sierro G, Cerretelli P. Muscle carnitine after strenuous endurance exercise. J Appl Physiol. 1992;72(2):423-7.

13. Dellavalle DM, Haas JD. Iron Supplementation Improves Energetic Efficiency in Iron-Depleted Female Rowers. Med Sci Sports Exerc. 2014;46(6):1204-15.

14. Desgorces FD, Chennaoui M, Gomez-Merino D, Drogou C, Guezennec CY. Leptin response to acute prolonged exercise after training in rowers. Eur J Appl Physiol. 2004;91(5-6):677-81.

15. Desgorces FD, Chennaoui M, Drogou C, Guezennec CY, Gomez-Merino D. Relationships between leptin levels and carbohydrate intake during rowing training. J Sports Med Phys Fitness. 2008;48(1):83-9.

16. Drenowatz C, Eisenmann JC, Carlson JJ, Pfeiffer KA, Pivarnik JM. Energy expenditure and dietary intake during high-volume and low-volume training periods among male endurance athletes. Appl Physiol Nutr Metab. 2012;37(2):199-205.

17. Drenowatz C, Eisenmann JC, Pivarnik JM, Pfeiffer KA, Carlson JJ. Differences in energy expenditure between high- and low-volume training. Eur J Sport Sci. 2013;13(4):422-30.

18. Emhoff CA, Messonnier LA, Horning MA, Fattor JA, Carlson TJ, Brooks GA. Gluconeogenesis and hepatic glycogenolysis during exercise at the lactate threshold. J Appl Physiol. 2013;114(3):297-306.

19. Enqvist JK, Mattsson CM, Johansson PH, Brink-Elfegoun T, Bakkman L, Ekblom BT. Energy turnover during 24 hours and 6 days of adventure racing. J Sports Sci. 2010;28(9):947-55.

20. Fudge BW, Westerterp KR, Kiplamai FK, Onywera VO, Boit MK, Kayser B et al. Evidence of negative energy balance using doubly labelled water in elite Kenyan endurance runners prior to competition. Br J Nutr. 2006;95(1):59-66.

21. Fudge BW, Easton C, Kingsmore D, Kiplamai FK, Onywera VO, Westerterp KR et al. Elite Kenyan Endurance Runners are Hydrated Day-To-Day with Ad Libitum Fluid Intake. Med Sci Sports Exerc. 2008;40(6):1171-9.

22. Garcia-Roves PM, Terrados N, Fernandez SF, Patterson AM. Macronutrients intake of top level cyclists during continuous competition--change in the feeding pattern. Int J Sports Med. 1998;19(1):61-7.

23. Garcia-Roves PM, Terrados N, Fernandez S, Patterson AM. Comparison of dietary intake and eating behavior of professional road cyclists during training and competition. Int J Sport Nutr Exerc Metab. 2000;10(1):82-98.

24. Gorsuch J, Long J, Miller K, Primeau K, Rutledge S, Sossong A et al. The effect of squat depth on multiarticular muscle activation in collegiate cross-country runners. J Strength Cond Res. 2013;27(9):2619-25.

25. Griffith RO, Dressendorfer RH, Fullbright GD, Wade CE. Testicular function during exhaustive endurance training. / La fonction testiculaire lors d ' un entrainement epuisant d ' endurance. Phys Sportsmed. 1990;18(5):54-6;61-2;4.

26. Hassapidou MN, Manstrantoni A. Dietary intakes of elite female athletes in Greece. J Hum Nutr Dietetics. 2001;14(5):391-6.

27. Havemann L, Goedecke JH. Nutritional practices of male cyclists before and during an ultraendurance event. Int J Sport Nutr Exerc Metab. 2008;18(6):551-66.

28. Heinonen A, Oja P, Kannus P, Sievanen H, Manttari A, Vuori I. Bone mineral density of female athletes in different sports. Bone Miner. 1993;23(1):1-14.

29. Herring JL, Mole PA, Meredith CN, Stern JS. Effect of suspending exercise training on resting metabolic rate in women. Med Sci Sports Exerc. 1992;24(1):59-65.

30. Hill RJ, Davies PS. Energy intake and energy expenditure in elite lightweight female rowers. Med Sci Sports Exerc. 2002;34(11):1823-9.

31. Hulton AT, Lahart I, Williams KL, Godfrey R, Charlesworth S, Wilson M et al. Energy expenditure in the Race Across America (RAAM). Int J Sports Med. 2010;31(7):463-7.

32. Jensen CD, Zaltas ES, Whittam JH. Dietary intakes of male endurance cyclists during training and racing. J Am Diet Assoc. 1992;92(8):986-8.

33. Jones PJ, Leitch CA. Validation of doubly labeled water for measurement of caloric expenditure in collegiate swimmers. J Appl Physiol. 1993;74(6):2909-14.

34. Jurimae J, Jurimae T, Pihl E. Rowing ergometer performance and anaerobic capacity in college rowers. Kinesiology. 1999;31(2):13-8.

35. Jurimae J, Hofmann P, Jurimae T, Maestu J, Purge P, Wonisch M et al. Plasma adiponectin response to sculling exercise at individual anaerobic threshold in college level male rowers. Int J Sports Med. 2006;27(4):272-7.

36. Jurimae J, Jurimae T. Plasma leptin responses to prolonged sculling in female rowers. J Sports Med Phys Fitness. 2004;44(1):104-9.

37. Jurimae J, Purge P, Jurimae T. Effect of prolonged training period on plasma adiponectin in elite male rowers. Horm Metab Res. 2007;39(7):519-23.

38. Jurimae J, Ramson R, Maestu J, Jurimae T, Arciero PJ, Braun WA et al. Interactions between adipose, bone, and muscle tissue markers during acute negative energy balance in male rowers. J Sports Med Phys Fitness. 2011;51(2):347-54.

39. Kabasakalis A, Kalitsis K, Tsalis G, Mougios V. Imbalanced nutrition of top-level swimmers. Int J Sports Med. 2007;28(9):780-6.

40. Koshimizu T, Matsushima Y, Yokota Y, Yanagisawa K, Nagai S, Okamura K et al. Basal metabolic rate and body composition of elite Japanese male athletes. J Med Invest. 2012;59(3-4):253-60.

41. LaForgia J, Withers RT, Williams AD, Murch BJ, Chatterton BE, Schultz CG et al. Effect of 3 weeks of detraining on the resting metabolic rate and body composition of trained males. Eur J Clin Nutr. 1999;53(2):126-33.

42. Lazzer S, Salvadego D, Rejc E, Buglione A, Antonutto G, di Prampero PE. The energetics of ultra-endurance running. Eur J Appl Physiol. 2012;112(5):1709-15.

43. Loftin M, Warren B, Mayhew J. Comparison of physiologic and performance variables in male and female cross-country runners during a competitive season. Sports Med Train Rehabil. 1992;3(4):281-8.

44. Maestu J, Jurimae J, Purge P, Ramson R, Jurimae T. Performance improvement is associated with higher postexercise responses in interleukin-6 and tumor necrosis factor concentrations. J Sports Med Phys Fitness. 2010;50(4):524-9.

45. Maïmoun, Lumbroso, Manetta, Paris, Leroux, Sultan. Testosterone is significantly reduced in endurance athletes without impact on bone mineral density. Horm Res. 2003;59(6):285-92.

46. Magkos F, Yannakoulia M, Kavouras SA, Sidossis LS. The type and intensity of exercise have independent and additive effects on bone mineral density. Int J Sports Med. 2007;28(9):773-9.

47. Margaritis I, Palazzetti S, Rousseau AS, Richard MJ, Favier A. Antioxidant supplementation and tapering exercise improve exercise-induced antioxidant response. J Am Coll Nutr. 2003;22(2):147-56.

48. Martin MK, Martin DT, Collier GR, Burke LM. Voluntary food intake by elite female cyclists during training and racing: influence of daily energy expenditure and body composition. Int J Sport Nutr Exerc Metab. 2002;12(3):249.

49. Medelli J, Lounana J, Menuet JJ, Shabani M, Cordero-MacIntyre Z. Is osteopenia a health risk in professional cyclists? J Clin Densitom. 2009;12(1):28-34.

50. Moses K, Manore MM. Development and testing of a carbohydrate monitoring tool for athletes. J Am Diet Assoc. 1991;91(8):962-5.

51. Motonaga K, Yoshida S, Yamagami F, Kawano T, Takeda E. Estimation of total daily energy expenditure and its components by monitoring the heart rate of Japanese endurance athletes. J Nutr Sci Vitaminol (Tokyo). 2006;52(5):360-7.

52. Muoio DM, Leddy JJ, Horvath PJ, Awad AB, Pendergast DR. Effect of dietary fat on metabolic adjustments to maximal VO2 and endurance in runners. Med Sci Sports Exerc. 1994;26(1):81-8.

53. Noland RC, Baker JT, Boudreau SR, Kobe RW, Tanner CJ, Hickner RC et al. Effect of intense training on plasma leptin in male and female swimmers. Med Sci Sports Exerc. 2001;33(2):227-31.

54. Ousley-Pahnke L, Black DR, Gretebeck RJ. Dietary intake and energy expenditure of female collegiate swimmers during decreased training prior to competition. J Am Diet Assoc. 2001;101(3):351-4.

55. Palazzetti S, Rousseau AS, Richard MJ, Favier A, Margaritis I. Antioxidant supplementation preserves antioxidant response in physical training and low antioxidant intake. Br J Nutr. 2004;91(1):91-100.

56. Palm R, Jürimäe J, Mästu J, Purge P, Jürimäe T, Rom K et al. Relationship between body composition and aerobic capacity values in well-trained male rowers. Acta Kinesiol Universitatis Tartu. 2005;10:125-32.

57. Papadopoulou SK, Gouvianaki A, Grammatikopoulou MG, Maraki Z, Pagkalos IG, Malliaropoulos N et al. Body composition and dietary intake of elite cross-country skiers members of the greek national team. Asian J Sports Med. 2012;3(4):257-66.

58. Penteado VS, Castro CH, Pinheiro Mde M, Santana M, Bertolino S, de Mello MT et al. Diet, body composition, and bone mass in well-trained cyclists. J Clin Densitom. 2010;13(1):43-50.

59. Peters EM, Goetzsche JM. Dietary practices of South African ultradistance runners. Int J Sport Nutr. 1997;7(2):80-103.

60. Phillips SM, Atkinson SA, Tarnopolsky MA, MacDougall JD. Gender differences in leucine kinetics and nitrogen balance in endurance athletes. J Appl Physiol (1985). 1993;75(5):2134-41.

61. Rehrer NJ, Hellemans IJ, Rolleston AK, Rush E, Miller BF. Energy intake and expenditure during a 6-day cycling stage race. Scand J Med Sci Sports. 2010;20(4):609-18.

62. Roberts D, Smith DJ. Training at moderate altitude: iron status of elite male swimmers. J Lab Clin Med. 1992;120(3):387-91.

63. Santos DA, Dawson JA, Matias CN, Rocha PM, Minderico CS, Allison DB et al. Reference values for body composition and anthropometric measurements in athletes. PLoS One. 2014;9(5):e97846.

64. Sato A, Shimoyama Y, Ishikawa T, Murayama N. Dietary thiamin and riboflavin intake and blood thiamin and riboflavin concentrations in college swimmers undergoing intensive training. Int J Sport Nutr Exerc Metab. 2011;21(3):195-204.

65. Schena F, Pattini A, Mantovanelli S. Iron status in athletes involved in endurance and in prevalently anaerobic sports. In: Kies CV, Driskell JA, editors. Sports nutrition: minerals and electrolytes. Boca Raton, FL: CRC Press; 1995. p. 65-80.

66. Schenk K, Gatterer H, Ferrari M, Ferrari P, Cascio VL, Burtscher M. Bike Transalp 2008: liquid intake and its effect on the body's fluid homeostasis in the course of a multistage, cross-country, MTB marathon race in the central Alps. Clin J Sport Med. 2010;20(1):47-52.

67. Schulz LO, Alger S, Harper I, Wilmore JH, Ravussin E. Energy expenditure of elite female runners measured by respiratory chamber and doubly labeled water. J Appl Physiol. 1992;72(1):23-8.

68. Sherman WM, Doyle JA, Lamb DR, Strauss RH. Dietary carbohydrate, muscle glycogen, and exercise performance during 7 d of training. Am J Clin Nutr. 1993;57(1):27-31.

69. Siders WA, Bolonchuk WW, Lukaski HC. Effects of participation in a collegiate sport season on body composition. J Sports Med Phys Fitness. 1991;31(4):571-6.

70. Siders WA, Lukaski HC, Bolonchuk WW. Relationships among swimming performance, body composition and somatotype in competitive collegiate swimmers. J Sports Med Phys Fitness. 1993;33(2):166-71.

71. Simsch C, Lormes W, Petersen KG, Baur S, Liu Y, Hackney AC et al. Training intensity influences leptin and thyroid hormones in highly trained rowers. Int J Sports Med. 2002;23(6):422-7.

72. Sjodin AM, Andersson AB, Hogberg JM, Westerterp KR. Energy balance in cross-country skiers: a study using doubly labeled water. Med Sci Sports Exerc. 1994;26(6):720-4.

73. Sundby OH, S. Gorelick ML. Relationship between functional hamstring: quadriceps ratios and running economy in highly trained and recreational female runners. J Strength Cond Res. 2014;28(8):2214-27.

74. Taylor SR, Rogers GG, Driver HS. Effects of training volume on sleep, psychological, and selected physiological profiles of elite female swimmers. Med Sci Sports Exerc. 1997;29(5):688-93.

75. Tomten SE, Hostmark AT. Energy balance in weight stable athletes with and without menstrual disorders. Scand J Med Sci Sports. 2006;16(2):127-33.

76. Trappe TA, Gastaldelli A, Jozsi AC, Troup JP, Wolfe RR. Energy expenditure of swimmers during high volume training. Med Sci Sports Exerc. 1997;29(7):950-4.

77. Vaiksaar S, Jurimae J, Maestu J, Purge P, Kalytka S, Shakhlina L et al. No effect of menstrual cycle phase on fuel oxidation during exercise in rowers. Eur J Appl Physiol. 2011;111(6):1027-34.

78. Winters KM, Adams WC, Meredith CN, Loan MD, Lasley BL. Bone density and cyclic ovarian function in trained runners and active controls. Med Sci Sports Exerc. 1996;28(7):776-85.

79. Witard OC, Jackman SR, Kies AK, Jeukendrup AE, Tipton KD. Effect of increased dietary protein on tolerance to intensified training. Med Sci Sports Exerc. 2011;43(4):598-607.

80. Yeater R, Reed C, Ullrich I, Morise A, Borsch M. Resistance trained athletes using or not using anabolic steroids compared to runners: effects on cardiorespiratory variables, body composition, and plasma lipids. Br J Sports Med. 1996;30(1):11-4.

81. Zajac A, Poprzecki S, Maszczyk A, Czuba M, Michalczyk M, Zydek G. The effects of a ketogenic diet on exercise metabolism and physical performance in off-road cyclists. Nutrients. 2014;6(7):2493-508.

82. Zalcman I, Guarita HV, Juzwiak CR, Crispim CA, Antunes HK, Edwards B et al. Nutritional status of adventure racers. Nutrition. 2007;23(5):404-11.

83. Downs SH, Black N. The feasibility of creating a checklist for the assessment of the methodological quality both of randomised and non-randomised studies of health care interventions. J Epidemiol Community Health. 1998;52(6):377-84.
