# Supplementary material for: Total Energy Expenditure, Energy Intake, and Body Composition in Endurance Athletes Across the Training Season: A Systematic Review
Source: Sports Med Open. 2017 Feb 4;3:8. doi: 10.1186/s40798-017-0076-1 (PMC5292109; doi:10.1186/s40798-017-0076-1)
Supplement: Additional file 3: — List of excluded references and reason for exclusion. (PDF 490 kb) [file 40798_2017_76_MOESM3_ESM.pdf]

Article title: Total Energy Expenditure, Energy Intake and Body Composition in Endurance Athletes across the Training Season: A Systematic Review.

Authors: Juliane Heydenreich, Bengt Kayser, Yves Schutz, Katarina Melzer

Journal name: Sports Medicine

Corresponding author: Juliane Heydenreich, Swiss Federal Institute of Sport, Hauptstrasse 247, 2532 Magglingen, Switzerland and University of Lausanne, Faculty of Biology and Medicine, Rue du Bugnon 21, 1011 Lausanne, Switzerland, e-mail: juliane.heydenreich@googlemail.com

Online Resource 4 – List of excluded references and reason for exclusion.

Reference	Seasonal phase NR	Inappropriate method	Age limit or NR	Multiple publications	No sex differentiation or NR	Case study	Data extraction not possible*	No habitual DI	No endurance athletes differentiation	EI, TEE during race
Almeras et al. 1997 [1]			x							
Andreoli et al. 2012 [2]			x							
Arciero et al. 1998 [3]					x					
Avlonitou et al. 1997 [4]	x									
Azevedo et al. 2007 [5]	x									
Baer 1993 [6]	x									
Baer & Taper 1992 [7]	x									
Baer et al. 1992 [8]	x									
Balaban et al. 1995 [9]			x							
Bale et al. 1996 [10]	x									
Ballor & Poehlman 1992 [11]	x									
Barr 1991 [12]	x									
Barrack et al. 2008 [13]				x						
Barrack et al. 2008 [14]			x							
Barrack et al. 2010 [15]			x							
Barrack et al. 2011 [16]	x									
Beidleman et al. 1995 [17]	x									
Beis et al. 2011 [18]					x					
Benson et al. 1990 [19]	x									
Black et al. 2012 [20]					x					

Brahm et al. 1997 [21]	x					
Brisswalter et al. 2011 [22]	x					
Brown & Cox 1998 [23]	x					
Brown et al. 2000 [24]				x		
Brown et al. 2009 [25]	x					
Bunt et al. 1990 [26]	x					
Buresh et al. 2005 [27]	x					
Burke et al. 2003 [28]				x		
Burrows et al. 2003 [29]	x					
Burtscher et al. 2010 [30]				x		
Campos et al. 2012 [31]				x		
Cardoso et al. 1994 [32]	x					
Carey 1992 [33]	x					
Carlsohn et al. 2011 [34]	x					
Casa et al. 2000 [35]	x					
Chia et al. 2013 [36]			x			
Christensen et al. 2002 [37]	x					
Claessens et al. 2000 [38]	x					
Cobb et al. 2003 [39]	x					
Constantini & Warren 1995 [40]		x				
Courteix et al. 1998 [41]			x			
Cox et al. 2010 [42]						x
da Costa et al. 2013 [43]	x					
Davy et al. 1996 [44]	x		x			
Deminice et al. 2010 [45]	x			x		
de Sousa et al. 2008 [46]	x					
Deutz et al. 2000 [47]	x					
Dias Quiterio et al. 2011 [48]	x					
Dumke et al. 2009 [49]	x					
Edwards et al. 1993 [50]	x					
Eisinger et al. 1994 [51]					x	
Elias et al. 1991 [52]	x					
Estok & Rudy 1994 [53]						
Ferguson et al. 2009 [54]				x		

Fiore et al. 1996 [55]	x							
FitzGerald & Carpenter 2010 [56]	x							
Fogelholm et al. 2000 [57]	x							
Frentsos & Baer 1997 [58]					x	x		
Frisch et al. 1993 [59]							x	
Gaine et al. 2007 [60]	x							
Gilbert et al. 1991 [61]	x							
Goedecke et al. 2000 [62]	x							
Gordon et al. 1996 [63]	x							
Greene et al. 2006 [64]	x							
Gremion et al. 2001 [65]	x							
Grund et al. 2001 [66]	x							
Guest & Barr 2005 [67]	x							
Hackett et al. 2013 [68]	x							
Hawley et al. 1997 [69]	x							
Hawley & Williams 1991 [70]	x							
Heinrich et al. 1990 [71]	x							
Hetland et al. 1993 [72]	x							
Hetland et al. 1995 [73]	x							
Herring et al. 1992 [74]	x							
Hind et al. 2011 [75]	x							
Hiscock & Mackinnon 1998 [76]							x	
Hoffman et al. 2010 [77]			x					
Hogstrom et al. 2012 [78]	x							
Hong et al. 2012 [79]	x							
Horton et al. 1994 [80]	x							x
Horvath et al. 2000 [81]	x							
Houltham & Rowlands 2014 [82]	x							
Hunter et al. 2011 [83]	x							
Hutchinson et al. 1991 [84]	x							
Jarvis et al. 2002 [85]	x							
Julian-Almarcegui et al. 2013 [86]	x							
Jurimae et al. 2007 [87]	x							
Jurimae & Jurimae 2001 [88]	x							

Jurimae & Jurimae 2004 [89]		x				
Jurimae et al. 2009 [90]	x					
Jurimae et al. 2010 [91]	x					
Jurimae et al. 2009 [92]			x			
Jurimae et al. 2011 [93]	x					
Karstoft et al. 2013 [94]				x		
Kimber et al. 2002 [95]						x
Klausen et al. 1993 [96]		x				
Knez & Peake 2010 [97]	x					
Koehler et al. 2010 [98]	x					
Koehler et al. 2011 [99]	x					
Kohrt et al. 1992 [100]	x					
Koury et al. 2004 [101]	x					
Koutedakis et al. 1994 [102]	x					
Kruseman et al. 2005 [103]				x		x
Laforgia et al. 1997 [104]	x					
Lampe et al. 1991 [105]	x					
Leake & Carter 1991 [106]					x	
Lebenstedt et al. 1999 [107]	x					
Leddy et al. 1997 [108]	x					
Lopez et al. 2000 [109]	x					
Lucas et al. 2003 [110]	x					
Luetkemeier & Thomas 1994 [111]	x					
Lukaski et al. 1990 [112]		x				
Lukaski et al. 1996 [113]		x				
Machefer et al. 2007 [114]		x				
Maestu et al. 2000 [115]	x					
Maestu et al. 2003 [116]					x	
Maimoun et al. 2013 [117]	x					
Maimoun et al. 2013 [118]	x					
Martinez et al. 2011 [119]		x				
Mauriege et al. 1997 [120]	x					
McClanahan et al. 2002 [121]					x	
Mendes et al. 2004 [122]				x		

Mieras et al. 2014 [123]	x				
Mitsuzono et al. 1995 [124]	x				
Mooses et al. 2013 [125]	x				
Morris et al. 1999 [126]	x				
Mueller et al. 2013 [127]		x			
Mulligan & Butterfield 1990 [128]	x				
Myerson et al. 1991 [129]	x				
Nichols et al. 2010 [130]	x				
Nieman et al. 2005 [131]	x				
Nieman et al 2012 [132]	x				
Nieves et al. 2010 [133]	x				
Nogueira & Da Costa 2004 [134]	x				
Nutter 1991 [135]	x				
Nuviala et al. 1999 [136]	x				
Nygren-Bonnier et al. 2007 [137]	x				
Ogawa et al. 1992 [138]	x				
Onywera et al. 2004 [139]			x		
Oosthuyse et al. 2013 [140]	x				
Ormsbee & Arciero 2012 [141]			x		
Ostachowska-Gasior et al. 2005 [142]	x				
Parizkova 1994 [143]		x			
Paschoal & Amancino 2004 [144]	x				
Pate et al. 1992 [145]	x				
Pate et al. 1990 [146]	x				
Penn et al. 1994 [147]	x				
Perry et al. 1992 [148]	x				
Petersen et al. 2006 [149]				x	
Petibois et al. 2004 [150]			x		
Pettersson et al. 1999 [151]	x				
Pettersson et al. 2000 [152]		x			
Pfeiffer et al. 2012 [153]					x
Pichard et al. 1997 [154]	x				
Popovic et al. 2010 [155]	x				
Poehlman et al. 1992 [156]	x				

Poehlman et al. 1990 [157]	x				
Procter-Gray et al. 2008 [158]	x				
Proctor et al. 1996 [159]	x				
Quinn et al. 2011 [160]		x			
Quintas et al. 2003 [161]	x				
Ratcliff et al. 2011 [162]	x				
Rector et al. 2009 [163]	x				
Rejc et al. 2010 [164]					x
Rickenlund et al. 2003 [165]	x				
Rico et al. 1993 [166]	x				
Rico et al. 1993 [167]	x				
Riddell et al. 2003 [168]	x				
Riley-Hagan et al. 1992 [169]	x				
Robertson et al. 1991 [170]	x				
Rogosheske et al. 1994 [171]	x				
Rokitzki et al. 1994 [172]	x				
Ross et al. 2014 [173]					x
Rossi et al. 2014 [174]				x	
Roy et al. 1998 [175]	x				
Rutherford 1993 [176]	x				
Ryan et al. 1996 [177]	x				
Sanchez-Benito et al. 2007 [178]	x				
Sanchez-Benito & Sanchez Soriano 2007 [179]	x				
Saunders et al. 1998 [180]	x			x	
Schmidt et al. 1994 [181]	x				
Schneider et al. 1992 [182]	x				
Schtscherbyna et al. 2009 [183]	x				
Schulz et al 1991 [184]	x				
Sherk et al. 2014 [185]	x				
Shimizu et al. 2012 [186]	x				
Shing et al. 2013 [187]				x	
Singh et al. 1990 [188]	x				
Singh et al. 1993 [189]				x	
Sjodin et al. 1996 [190]			x		

Skarda & Burge 1998 [191]	x					
Slater et al 2005 [192]				x		
Slattery et al. 2012 [193]				x		
Smathers et al. 2009 [194]	x					
Smith & Rutherford 1993 [195]	x					
Snead et al. 1992 [196]	x					
Snow et al. 2000 [197]	x					
Soot et al. 2006 [198]	x					
Soot et al. 2007 [199]	x					
Soria et a. 2011 [200]	x					
Soultanakis & Platanou 2008 [201]		x				
Stewart & Hannan 2000 [202]	x					
Stöggl et al. 2013 [203]	x					
Stuempfle et al. 2002 [204]						x
Stuempfle et al. 2003 [205]						x
Stuempfle et al. 2013 [206]						x
Stray-Gundersen et al. 1991 [207]		x				
Strid et al. 2011 [208]				x		
Sudi et al. 2001 [209]	x					
Suetta et al. 1996 [210]	x					
Sutherland et al. 1991 [211]	x					
Sutherland et al. 1992 [212]	x					
Syrotuik et al. 2001 [213]				x		
Taaffe & Marcus 1999 [214]	x					
Taaffe et al. 1995 [215]	x					
Taipale et al. 2014 [216]					x	
Tarnopolsky et al. 1995 [217]	x					
Tarnopolsky et al. 1997 [218]	x					
Thompson et al. 1993 [219]	x					
Thompson et al. 1995 [220]	x					
Thompson & Manore 1996 [221]	x					
Tomten et al. 1998 [222]	x					
Tomten & Hostmark 2009 [223]			x			
Tremblay et al. 1990 [224]	x					

Tuuri et al. 2002 [225]	x	x		
Vanheest et al. 2014 [226]		x		
Vogt et al. 2005 [227]			x	
Warren et al. 1990 [228]	x			
Weight et al. 1991 [229]	x			
Weight et al. 1992 [230]	x			
Weitkunat et al. 2012 [231]				x
Wesche & Wiersinga 2001 [232]			x	
Wilber et al. 1996 [233]	x			
Williams et al. 1992 [234]			x	
Williford et al. 1993 [235]	x			
Wilmore et a. 1992 [236]	x			
Withers et al. 1992 [237]	x			
Withers et al. 1993 [238]	x			
Wulff Helge et al. 2012 [239]	x			
Zaccaria et al. 2013 [240]	x			
Zietz et al. 2009 [241]		x		

*e.g. no absolute values reported, no values for energy/macronutrient intake reported

NR = not reported

References

1. Almeras N, Lemieux S, Bouchard C, Tremblay A. Fat gain in female swimmers. Physiol Behav. 1997;61(6):811-7.

2. Andreoli A, Marfe G, Manzi V, Sinibaldi-Salimei P. Is body cell mass a predictive index of performance in male recreational long-distance runners? Sport Sci Health. 2012;8(1):47-50.

3. Arciero PJ, Smith DL, Calles-Escandon J. Effects of short-term inactivity on glucose tolerance, energy expenditure, and blood flow in trained subjects. J Appl Physiol (1985). 1998;84(4):1365-73.

4. Avlonitou E, Georgiou E, Douskas G, Louizi A. Estimation of body composition in competitive swimmers by means of three different techniques. Int J Sports Med. 1997;18(5):363-8.

5. Azevedo JL, Tietz E, Two-Feathers T, Paull J, Chapman K. Lactate, fructose and glucose oxidation profiles in sports drinks and the effect on exercise performance. PLoS One. 2007;2(9):e927.

6. Baer JT. Endocrine parameters in amenorrheic and eumenorrheic adolescent female runners. Int J Sports Med. 1993;14(4):191-5.

7. Baer JT, Taper LJ. Amenorrheic and eumenorrheic adolescent runners: dietary intake and exercise training status. J Am Diet Assoc. 1992;92:89-91.

8. Baer JT, Taper LJ, Gwazdauskas FG, Walberg JL, Novascone MA, Ritchey SJ et al. Diet, hormonal, and metabolic factors affecting bone mineral density in adolescent amenorrheic and eumenorrheic female runners. J Sports Med Phys Fitness. 1992;32(1):51-8.

9. Balaban EP, Snell P, Stray-Gundersen J, Frenkel EP. The effect of running on serum and red cell ferritin. A longitudinal comparison. Int J Sports Med. 1995;16(5):278-82.

10. Bale P, Doust J, Dawson D. Gymnasts, distance runners, anorexics body composition and menstrual status. J Sports Med Phys Fitness. 1996;36(1):49-53.

11. Ballor DL, Poehlman ET. Resting metabolic rate and coronary-heart-disease risk factors in aerobically and resistance-trained women. Am J Clin Nutr. 1992;56(6):968-74.

12. Barr SI. Relationship of eating attitudes to anthropometric variable and dietary intakes of female collegiate swimmers. J Am Diet Assoc. 1991;91(8):976-7.

13. Barrack MT, Rauh MJ, Barkai HS, Nichols JF. Dietary restraint and low bone mass in female adolescent endurance runners. Am J Clin Nutr. 2008;87(1):36-43.

14. Barrack MT, Rauh MJ, Nichols JF. Prevalence of and traits associated with low BMD among female adolescent runners. Med Sci Sports Exerc. 2008;40(12):2015-21.

15. Barrack MT, Van Loan MD, Rauh MJ, Nichols JF. Physiologic and behavioral indicators of energy deficiency in female adolescent runners with elevated bone turnover. Am J Clin Nutr. 2010;92(3):652-9.

16. Barrack MT, Von Loan MD, Rauh MJ, Nichols JF. Body Mass, Training, Menses, and Bone in Adolescent Runners: A 3-yr Follow-up. Med Sci Sports Exerc. 2011;43(6):959-66.

17. Beidleman BA, Puhl JL, De Souza MJ. Energy balance in female distance runners. Am J Clin Nutr. 1995;61(2):303-11.

18. Beis LY, Willkomm L, Ross R, Bekele Z, Wolde B, Fudge B et al. Food and macronutrient intake of elite Ethiopian distance runners. J Int Soc Sports Nutr. 2011;8(1):7-13.

19. Benson JE, Allemann Y, Theintz GE, Howald H. Eating problems and calorie intake levels in Swiss adolescent athletes. International Journal of Sports Medicine. 1990;11(4):249-52.

20. Black KE, Skidmore PM, Brown RC. Energy intakes of ultraendurance cyclists during competition, an observational study. Int J Sport Nutr Exerc Metab. 2012;22(1):19-23.

21. Brahm H, Strom H, Piehl-Aulin K, Mallmin H, Ljunghall S. Bone metabolism in endurance trained athletes: a comparison to population-based controls based on DXA, SXA, quantitative ultrasound, and biochemical markers. Calcif Tissue Int. 1997;61(6):448-54.

22. Brisswalter J, Bouhlel E, Falola JM, Abbiss CR, Vallier JM, Hauswirth C. Effects of Ramadan Intermittent Fasting on Middle-Distance Running Performance in Well-Trained Runners. Clin J Sport Med. 2011;21(5):422-7.

23. Brown RC, Cox CM. Effects of high fat versus high carbohydrate diets on plasma lipids and lipoproteins in endurance athletes. Med Sci Sports Exerc. 1998;30(12):1677-83.

24. Brown RC, Cox CM, Goulding A. High-carbohydrate versus high-fat diets: effect on body composition in trained cyclists. Med Sci Sports Exerc. 2000;32(3):690-4.

25. Brown MB, Millard-Stafford ML, Allison AR. Running-specific prostheses permit energy cost similar to nonamputees. Med Sci Sports Exerc. 2009;41(5):1080-7.

26. Bunt JC, Going SB, Lohman TG, Heinrich CH, Perry CD, Pamenter RW. Variation in bone mineral content and estimated body fat in young adult females. Med Sci Sports Exerc. 1990;22(5):564-9.

27. Buresh R, Berg K, Noble J. Heat Production and Storage Are Positively Correlated With Measures of Body Size/Composition and Heart Rate Drift During Vigorous Running. Res Q Exerc Sport. 2005;76(3):267-74.

28. Burke LM, Slater G, Broad EM, Haukka J, Modulon S, Hopkins WG. Eating patterns and meal frequency of elite Australian athletes. Int J Sport Nutr Exerc Metab. 2003;13(4):521-38.

29. Burrows M, Nevill AM, Bird S, Simpson D. Physiological factors associated with low bone mineral density in female endurance runners. Br J Sports Med. 2003;37(1):67.

30. Burtscher M, Gatterer H, Faulhaber M, Gerstgrasser W, Schenk K. Effects of Intermittent Hypoxia on Running Economy. International Journal of Sports Medicine. 2010;31(9):644-50.

31. Campos EZ, Bastos FN, Papoti M, Freitas Junior IF, Gobatto CA, Balikian Junior P. The effects of physical fitness and body composition on oxygen consumption and heart rate recovery after high-intensity exercise. Int J Sports Med. 2012;33(8):621-6.

32. Cardoso GC, Posadas C, Orvananos OO, Peniche C, Zamora J, Aguilar R et al. Long distance runners and body-builders exhibit elevated plasma levels of lipoprotein(a). Chem Phys Lipids. 1994;67-68:207-21.

33. Carey GB. A nutrition guidebook to improve the diet of male and female collegiate swimmers. J Swimming Res. 1992;8:24-9.

34. Carlsohn A, Scharhag-Rosenberger F, Cassel M, Mayer F. Resting metabolic rate in elite rowers and canoeists: difference between indirect calorimetry and prediction. Ann Nutr Metab. 2011;58(3):239-44.

35. Casa DJ, Maresh CM, Armstrong LE, Kavouras SA, Herrera JA, Hacker FT, Jr. et al. Intravenous versus oral rehydration during a brief period: responses to subsequent exercise in the heat. Med Sci Sports Exerc. 2000;32(1):124-33.

36. Chia M, Liao CA, Huang CY, Lee WC, Hou CW, Yu SH et al. Reducing body fat with altitude hypoxia training in swimmers: role of blood perfusion to skeletal muscles. Chin J Physiol. 2013;56(1):18-25.

37. Christensen DL, Van Hall G, Hambraeus L. Food and macronutrient intake of male adolescent Kalenjin runners in Kenya. Br J Nutr. 2002;88(6):711-7.

38. Claessens M, Claessens C, Claessens P, Henderieckx J, Claessens J. Importance of determining the percentage body fat in endurance-trained athletes. Indian Heart J. 2000;52(3):307-14.

39. Cobb KL, Bachrach LK, Greendale G, Marcus R, Neer RM, Nieves J et al. Disordered eating, menstrual irregularity, and bone mineral density in female runners. Med Sci Sports Exerc. 2003;35(5):711-9.

40. Constantini NW, Warren MP. Menstrual dysfunction in swimmers: a distinct entity. J Clin Endocrinol Metab. 1995;80(9):2740-4.

41. Courteix D, Lespessailles E, Peres SL, Obert P, Germain P, Benhamou CL. Effect of physical training on bone mineral density in prepubertal girls: a comparative study between impact-loading and non-impact-loading sports. Osteoporos Int. 1998;8(2):152-8.

42. Cox GR, Snow RJ, Burke LM. Race-Day Carbohydrate Intakes of Elite Triathletes Contesting Olympic-Distance Triathlon Events. International Journal of Sport Nutrition & Exercise Metabolism. 2010;20(4):299-306.

43. da Costa NF, Schtscherbyna A, Soares EA, Ribeiro BG. Disordered eating among adolescent female swimmers: dietary, biochemical, and body composition factors. Nutrition. 2013;29(1):172-7.

44. Davy KP, Evans SL, Stevenson ET, Seals DR. Adiposity and regional body fat distribution in physically active young and middle-aged women. Int J Obes Relat Metab Disord. 1996;20(8):777-83.

45. Deminice R, Trindade CS, Degiovanni GC, Garlip MR, Portari GV, Teixeira M et al. Oxidative stress biomarkers response to high intensity interval training and relation to performance in competitive swimmers. J Sports Med Phys Fitness. 2010;50(3):356-62.

46. de Sousa EF, Da Costa TH, Nogueira JA, Vivaldi LJ. Assessment of nutrient and water intake among adolescents from sports federations in the Federal District, Brazil. Br J Nutr. 2008;99(6):1275-83.

47. Deutz RC, Benardot D, Martin DE, Cody MM. Relationship between energy deficits and body composition in elite female gymnasts and runners. Med Sci Sports Exerc. 2000;32(3):659-68.

48. Dias Quiterio AL, Carnero EA, Baptista FM, Sardinha LB. Skeletal mass in adolescent male athletes and nonathletes: relationships with high-impact sports. J Strength Cond Res. 2011;25(12):3439-47.

49. Dumke CL, Nieman DC, Utter AC, Rigby MD, Quindry JC, Triplett NT et al. Quercetin's effect on cycling efficiency and substrate utilization. Appl Physiol Nutr Metab. 2009;34(6):993-1000.

50. Edwards JE, Lindeman AK, Mikesky AE, Stager JM. Energy balance in highly trained female endurance runners. Med Sci Sports Exerc. 1993;25(12):1398-404.

51. Eisinger M, Plath M, Jung K, Leitzmann C. Nutrient intake of endurance runners with ovo-lacto-vegetarian diet and regular western diet. Z Ernahrungswiss. 1994;33(3):217-29.

52. Elias BA, Berg KE, Latin RW, Mellion MB, Hofschire PJ. Cardiac structure and function in weight trainers, runners, and runner/weight trainers. Res Q Exerc Sport. 1991;62(3):326-32.

53. Estok PJ, Rudy EB. Nutrient intake of women runners and nonrunners. Health Care Women Int. 1994;15(5):435-51.

54. Ferguson LM, Rossi KA, Ward E, Jadwin E, Miller TA, Miller WC. Effects of caloric restriction and overnight fasting on cycling endurance performance. J Strength Cond Res. 2009;23(2):560-70.

55. Fiore CE, Dieli M, Vintaloro G, Gibilaro M, Giacone G, Cottini E. Body composition and bone mineral density in competitive athletes in different sports. Int J Tissue React. 1996;18(4-6):121-4.

56. FitzGerald L, Carpenter C. Bone mineral density results influencing health-related behaviors in male athletes at risk for osteoporosis. J Clin Densitom. 2010;13(3):256-62.

57. Fogelholm M, Rankinen T, Isokaanta M, Kujala U, Uusitupa M. Growth, dietary intake, and trace element status in pubescent athletes and schoolchildren. Med Sci Sports Exerc. 2000;32(4):738-46.

58. Frentsos JA, Baer JT. Increased energy and nutrient intake during training and competition improves elite triathletes' endurance performance. Int J Sport Nutr. 1997;7(1):61-71.

59. Frisch RE, Snow RC, Johnson LA, Gerard B, Barbieri R, Rosen B. Magnetic resonance imaging of overall and regional body fat, estrogen metabolism, and ovulation of athletes compared to controls. J Clin Endocrinol Metab. 1993;77(2):471-7.

60. Gaine PC, Pikosky MA, Bolster DR, Martin WF, Maresh CM, Rodriguez NR. Postexercise whole-body protein turnover response to three levels of protein intake. Med Sci Sports Exerc. 2007;39(3):480-6.

61. Gilbert JA, Misner JE, Boileau RA, Ji LL, Slaughter MH. Lower thermic effect of a meal post-exercise in aerobically trained and resistance-trained subjects. Med Sci Sports Exerc. 1991;23(7):825-30.

62. Goedecke JH, St Clair Gibson A, Grobler L, Collins M, Noakes TD, Lambert EV. Determinants of the variability in respiratory exchange ratio at rest and during exercise in trained athletes. Am J Physiol Endocrinol Metab. 2000;279(6):E1325-34.

63. Gordon PM, Visich PS, Goss FL, Fowler S, Warty V, Denys BJ et al. Comparison of exercise and normal variability on HDL cholesterol concentrations and lipolytic activity. Int J Sports Med. 1996;17(5):332-7.

64. Greene DA, Naughton GA, Briody JN, Kemp A, Woodhead H. Assessment of bone strength at differentially-loaded skeletal regions in adolescent middle-distance runners. J Sci Med Sport. 2006;9(3):221-30.

65. Gremion G, Rizzoli R, Slosman D, Theintz G, Bonjour JP. Oligo-amenorrheic long-distance runners may lose more bone in spine than in femur. / Des coureuses de fond oligo-amenorrheiques peuvent perdre plus de masse osseuse au niveau de la colonne vertebrale qu'au niveau du femur. Medicine & Science in Sports & Exercise. 2001;33(1):15-21.

66. Grund A, Krause H, Kraus M, Siewers M, Rieckert H, Muller MJ. Association between different attributes of physical activity and fat mass in untrained, endurance- and resistance-trained men. Eur J Appl Physiol. 2001;84(4):310-20.

67. Guest NS, Barr SI. Cognitive Dietary Restraint Is Associated with Stress Fractures in Women Runners. Int J Sport Nutr Exerc Metab. 2005;15(2):147-59.

68. Hackett DA, Johnson N, Chow C. Respiratory muscle adaptations: a comparison between bodybuilders and endurance athletes. J Sports Med Phys Fitness. 2013;53(2):139-45.

69. Hawley JA, Palmer GS, Noakes TD. Effects of 3 days of carbohydrate supplementation on muscle glycogen content and utilisation during a 1-h cycling performance. Eur J Appl Physiol Occup Physiol. 1997;75(5):407-12.

70. Hawley JA, Williams MM. Dietary intakes of age-group swimmers. Br J Sports Med. 1991;25(3):154-8.

71. Heinrich CH, Going SB, Pamenter RW, Perry CD, Boyden TW, Lohman TG. Bone mineral content of cyclically menstruating female resistance and endurance trained athletes. Med Sci Sports Exerc. 1990;22(5):558-63.

72. Hetland ML, Haarbo J, Christiansen C. Low bone mass and high bone turnover in male long distance runners. J Clin Endocrinol Metab. 1993;77(3):770-5.

73. Hetland ML, Haarbo J, Christiansen C. Body composition and serum lipids in female runners: influence of exercise level and menstrual bleeding pattern. Eur J Clin Invest. 1995;25(8):553-8.

74. Herring JL, Mole PA, Meredith CN, Stern JS. Effect of suspending exercise training on resting metabolic rate in women. Med Sci Sports Exerc. 1992;24(1):59-65.

75. Hind K, Zanker C, Truscott J. Five-year follow-up investigation of bone mineral density by age in premenopausal elite-level long-distance runners. Clin J Sport Med. 2011;21(6):521-3.

76. Hiscock N, Mackinnon LT. A comparison of plasma glutamine concentration in athletes from different sports. Med Sci Sports Exerc. 1998;30(12):1693-6.

77. Hoffman MD, Lebus DK, Ganong AC, Casazza GA, Van Loan M. Body composition of 161-km ultramarathoners. Int J Sports Med. 2010;31(2):106-9.

78. Hogstrom GM, Pietila T, Nordstrom P, Nordstrom A. Body composition and performance: influence of sport and gender among adolescents. J Strength Cond Res. 2012;26(7):1799-804.

79. Hong K, Kim K, Lee S. High percentage of fat intakes, not low fat oxidation, may induce overweight cyclists. J Sports Med Phys Fitness. 2012;52(4):405-12.

80. Horton TJ, Drougas HJ, Sharp TA, Martinez LR, Reed GW, Hill JO. Energy balance in endurance-trained female cyclists and untrained controls. Journal of Applied Physiology. 1994;76(5):1937-45.

81. Horvath PJ, Eagen CK, Fisher NM, Leddy JJ, Pendergast DR. The effects of varying dietary fat on performance and metabolism in trained male and female runners. J Am Coll Nutr. 2000;19(1):52-60.

82. Houltham SD, Rowlands DS. A snapshot of nitrogen balance in endurance-trained women. Appl Physiol Nutr Metab. 2014;39(2):219-25.

83. Hunter GR, Katsoulis K, McCarthy JP, Ogard WK, Bamman MM, Wood DS et al. Tendon length and joint flexibility are related to running economy. Med Sci Sports Exerc. 2011;43(8):1492-9.

84. Hutchinson PL, Cureton KJ, Outz H, Wilson G. Relationship of cardiac size to maximal oxygen uptake and body size in men and women. Int J Sports Med. 1991;12(4):369-73.

85. Jarvis M, McNaughton L, Seddon A, Thompson D. The acute 1-week effects of the Zone Diet on body composition, blood lipid levels, and performance in recreational endurance athletes. J Strength Cond Res. 2002;16(1):50-7.

86. Julian-Almarcegui C, Gomez-Cabello A, Gonzalez-Aguero A, Olmedillas H, Gomez-Bruton A, Matute-Llorente A et al. The nutritional status in adolescent Spanish cyclists. Nutr Hosp. 2013;28(4):1184-9.

87. Jurimae J, Cicchella A, Jurimae T, Latt E, Haljaste K, Purge P et al. Regular physical activity influences plasma ghrelin concentration in adolescent girls. Med Sci Sports Exerc. 2007;39(10):1736-41.

88. Jurimae J, Jurimae T. Body composition analysis by bioelectrical impedance: effect of single training session in male rowers. Papers Anthropol. 2001;10:67-72.

89. Jurimae J, Jurimae T. EFFECT OF DIFFERENT PHYSICAL ACTIVITY ON BONE MINERAL DENSITY IN PUBERTAL GIRLS: THE ROLE OF BODY COMPOSITIONAL AND MUSCLE PERFORMANCE PARAMETERS. Papers on Anthropology. 2004;13:56-61.

90. Jurimae J, Latt E, Haljaste K, Purge P, Cicchella A, Jurimae T. Influence of puberty on ghrelin and BMD in athletes. Int J Sports Med. 2009;30(6):403-7.

91. Jurimae J, Latt E, Haljaste K, Purge P, Cicchella A, Jurimae T. A longitudinal assessment of ghrelin and bone mineral density with advancing pubertal maturation in adolescent female athletes. J Sports Med Phys Fitness. 2010;50(3):343-9.

92. Jurimae J, Ramson R, Maestu J, Purge P, Jurimae T, Arciero PJ et al. Plasma visfatin and ghrelin response to prolonged sculling in competitive male rowers. Med Sci Sports Exerc. 2009;41(1):137-43.

93. Jurimae J, Vaiksaar S, Maestu J, Purge P, Jurimae T. Adiponectin and bone metabolism markers in female rowers: eumenorrheic and oral contraceptive users. J Endocrinol Invest. 2011;34(11):835-9.

94. Karstoft K, Solomon TP, Laye MJ, Pedersen BK. Daily marathon running for a week--the biochemical and body compositional effects of participation. J Strength Cond Res. 2013;27(11):2927-33.

95. Kimber NE, Ross JJ, Mason SL, Speedy DB. Energy Balance During an Ironman Triathlon in Male and Female Triathletes. International Journal of Sport Nutrition & Exercise Metabolism. 2002;12(1):47.

96. Klausen T, Breum L, Fogh-Andersen N, Bennett P, Hippe E. The effect of short and long duration exercise on serum erythropoietin concentrations. Eur J Appl Physiol Occup Physiol. 1993;67(3):213-7.

97. Knez WL, Peake JM. The Prevalence of Vitamin Supplementation in Ultraendurance Triathletes. International Journal of Sport Nutrition & Exercise Metabolism. 2010;20(6):507-14.

98. Koehler K, Braun H, De Marees M, Fusch G, Fusch C, Mester J et al. Parallel assessment of nutrition and activity in athletes: Validation against doubly labelled water, 24-h urea excretion, and indirect calorimetry. Journal of Sports Sciences. 2010;28(13):1435-49.

99. Koehler K, Braun H, de Marees M, Fusch G, Fusch C, Schaenzer W. Assessing energy expenditure in male endurance athletes: validity of the SenseWear Armband. Med Sci Sports Exerc. 2011;43(7):1328-33.

100. Kohrt WM, Malley MT, Dalsky GP, Holloszy JO. Body composition of healthy sedentary and trained, young and older men and women. Med Sci Sports Exerc. 1992;24(7):832-7.

101. Koury JC, de Olilveria AV, Jr., Portella ES, de Olilveria CF, Lopes GC, Donangelo CM. Zinc and copper biochemical indices of antioxidant status in elite athletes of different modalities. Int J Sport Nutr Exerc Metab. 2004;14(3):358-72.

102. Koutedakis Y, Pacy PJ, Quevedo RM, Millward DJ, Hesp R, Boreham C et al. The effects of two different periods of weight-reduction on selected performance parameters in elite lightweight oarswomen. Int J Sports Med. 1994;15(8):472-7.

103. Kruseman M, Bucher S, Bovard M, Kayser B, Bovier PA. Nutrient intake and performance during a mountain marathon: an observational study. Eur J Appl Physiol. 2005;94(1-2):151-7.

104. Laforgia J, Withers RT, Shipp NJ, Gore CJ. Comparison of energy expenditure elevations after submaximal and supramaximal running. J Appl Physiol (1985). 1997;82(2):661-6.

105. Lampe JW, Slavin JL, Apple FS. Iron status of active women and the effect of running a marathon on bowel function and gastrointestinal blood loss. Int J Sports Med. 1991;12(2):173-9.

106. Leake CN, Carter JE. Comparison of body composition and somatotype of trained female triathletes. J Sports Sci. 1991;9(2):125-35.

107. Lebenstedt M, Platte P, Pirke KM. Reduced resting metabolic rate in athletes with menstrual disorders. Med Sci Sports Exerc. 1999;31(9):1250-6.

108. Leddy J, Horvath P, Rowland J, Pendergast D. Effect of a high or a low fat diet on cardiovascular risk factors in male and female runners. Med Sci Sports Exerc. 1997;29(1):17-25.

109. Lopez P, Ledoux M, Garrel DR. Increased thermogenic response to food and fat oxidation in female athletes: relationship with VO(2 max). Am J Physiol Endocrinol Metab. 2000;279(3):E601-7.

110. Lucas JA, Lucas PR, Vogel S, Gamble GD, Evans MC, Reid IR. Effect of sub-elite competitive running on bone density, body composition and sexual maturity of adolescent females. Osteoporos Int. 2003;14(10):848-56.

111. Luetkemeier MJ, Thomas EL. Hypervolemia and cycling time trial performance. Med Sci Sports Exerc. 1994;26(4):503-9.

112. Lukaski HC, Hoverson BS, Gallagher SK, Bolonchuk WW. Physical training and copper, iron, and zinc status of swimmers. Am J Clin Nutr. 1990;51(6):1093-9.

113. Lukaski HC, Siders WA, Hoverson BS, Gallagher SK. Iron, copper, magnesium and zinc status as predictors of swimming performance. Int J Sports Med. 1996;17(7):535-40.

114. Machefer G, Groussard C, Zouhal H, Vincent S, Youssef H, Faure H et al. Nutritional and plasmatic antioxidant vitamins status of ultra endurance athletes. J Am Coll Nutr. 2007;26(4):311-6.

115. Maestu J, Jurimae J, Jurimae T. Relationships between anthropometric variables and different rowing ergometer tests in heavyweight and lightweight male rowers. Papers on Anthropology. 2000;9:125-32.

116. Maestu J, Jurimae J, Jurimae T. Hormonal reactions during heavy training stress and following tapering in highly trained male rowers. Horm Metab Res. 2003;35(2):109-13.

117. Maimoun L, Coste O, Mura T, Philibert P, Galtier F, Mariano-Goulart D et al. Specific bone mass acquisition in elite female athletes. J Clin Endocrinol Metab. 2013;98(7):2844-53.

118. Maimoun L, Coste O, Philibert P, Briot K, Mura T, Galtier F et al. Peripubertal female athletes in high-impact sports show improved bone mass acquisition and bone geometry. Metab. 2013;62(8):1088-98.

119. Martinez S, Pasquarelli BN, Romaguera D, Arasa C, Tauler P, Aguilo A. Anthropometric characteristics and nutritional profile of young amateur swimmers. J Strength Cond Res. 2011;25(4):1126-33.

120. Mauriege P, Prud'Homme D, Marcotte M, Yoshioka M, Tremblay A, Despres JP. Regional differences in adipose tissue metabolism between sedentary and endurance-trained women. Am J Physiol. 1997;273(3 Pt 1):E497-506.

121. McClanahan BS, Ward KD, Vukadinovich C, Klesges RC, Chitwood L, Kinzey SJ et al. Bone mineral density in triathletes over a competitive season. J Sports Sci. 2002;20(6):463-9.

122. Mendes RR, Pires I, Oliveira A, Tirapegui J. Effects of creatine supplementation on the performance and body composition of competitive swimmers. J Nutr Biochem. 2004;15(8):473-8.

123. Mieras ME, S. Heesch MW, Slivka DR. Physiological and psychological responses to outdoor vs. laboratory cycling. J Strength Cond Res. 2014;28(8):2324-9.

124. Mitsuzono R, Komiya S, Migita T. Validity of total body water assessment for determining changes of body composition during endurance training in distance runners. Appl Human Sci. 1995;14(6):303-4.

125. Mooses M, Jurimae J, Maestu J, Mooses K, Purge P, Jurimae T. Running economy and body composition between competitive and recreational level distance runners. Acta Physiol Hung. 2013;100(3):340-6.

126. Morris FL, Payne WR, Wark JD. The impact of intense training on endogenous estrogen and progesterone concentrations and bone mineral acquisition in adolescent rowers. Osteoporos Int. 1999;10(5):361-8.

127. Mueller SM, Anliker E, Knechtle P, Knechtle B, Toigo M. Changes in body composition in triathletes during an Ironman race. Eur J Appl Physiol. 2013;113(9):2343-52.

128. Mulligan K, Butterfield GE. Discrepancies between energy intake and expenditure in physically active women. Br J Nutr. 1990;64(1):23-36.

129. Myerson M, Gutin B, Warren MP, May MT, Contento I, Lee M et al. Resting metabolic rate and energy balance in amenorrheic and eumenorrheic runners. Med Sci Sports Exerc. 1991;23(1):15-22.

130. Nichols JF, Aralis H, Merino SG, Barrack MT, Stalker-Fader L, Rauh MJ. Utility of the actiheart accelerometer for estimating exercise energy expenditure in female adolescent runners. Int J Sport Nutr Exerc Metab. 2010;20(6):487-95.

131. Nieman DC, Davis JM, Henson DA, Gross SJ, Dumke CL, Utter AC et al. Muscle cytokine mRNA changes after 2.5 h of cycling: influence of carbohydrate. Med Sci Sports Exerc. 2005;37(8):1283-90.

132. Nieman DC, Konrad M, Henson DA, Kennerly K, Shanely RA, Wallner-Liebmann SJ. Variance in the acute inflammatory response to prolonged cycling is linked to exercise intensity. J Interferon Cytokine Res. 2012;32(1):12-7.

133. Nieves JW, Melsop K, Curtis M, Kelsey JL, Bachrach LK, Greendale G et al. Nutritional factors that influence change in bone density and stress fracture risk among young female cross-country runners. PM R. 2010;2(8):740-50; quiz 94.

134. Nogueira JA, Da Costa TH. Nutrient intake and eating habits of triathletes on a Brazilian diet. Int J Sport Nutr Exerc Metab. 2004;14(6):684-97.

135. Nutter J. Relationship of selected body composition and training variables to 17 B-estradiol in eumenorrheic runners. J Sports Med Phys Fitness. 1991;31(3):396-400.

136. Nuviala RJ, Lapieza MG, Bernal E. Magnesium, zinc, and copper status in women involved in different sports. Int J Sport Nutr. 1999;9(3):295-309.

137. Nygren-Bonnier M, Gullstrand L, Klefbeck B, Lindholm P. Effects of glossopharyngeal pistoning for lung insufflation in elite swimmers. Med Sci Sports Exerc. 2007;39(5):836-41.

138. Ogawa T, Spina RJ, Martin WH, 3rd, Kohrt WM, Schechtman KB, Holloszy JO et al. Effects of aging, sex, and physical training on cardiovascular responses to exercise. Circulation. 1992;86(2):494-503.

139. Onywera VO, Kiplamai FK, Boit MK, Pitsiladis YP. Food and macronutrient intake of elite kenyan distance runners. Int J Sport Nutr Exerc Metab. 2004;14(6):709-19.

140. Oosthuyse T, Viedge A, McVeigh J, Avidon I. Anaerobic power in road cyclists is improved after 10 weeks of whole-body vibration training. J Strength Cond Res. 2013;27(2):485-94.

141. Ormsbee MJ, Arciero PJ. Detraining increases body fat and weight and decreases VO2peak and metabolic rate. J Strength Cond Res. 2012;26(8):2087-95.

142. Ostachowska-Gasior A, Kolarzyk E, Szot W, Lyszczarz J. The relation between antioxidative ability and the diet of young swimmers. Rocz Akad Med Bialymst. 2005;50 Suppl 1:241-4.

143. Parizkova J. Dietary intake and body physique in adolescent cross-country skiers. J Sports Sci. 1994;12(3):251-4.

144. Paschoal VC, Amancio OM. Nutritional status of Brazilian elite swimmers. Int J Sport Nutr Exerc Metab. 2004;14(1):81-94.

145. Pate RR, Macera CA, Bailey SP, Bartoli WP, Powell KE. Physiological, anthropometric, and training correlates of running economy. Med Sci Sports Exerc. 1992;24(10):1128-33.

146. Pate RR, Sargent RG, Baldwin C, Burgess ML. Dietary intake of women runners. Int J Sports Med. 1990;11(6):461-6.

147. Penn IW, Wang ZM, Buhl KM, Allison DB, Burastero SE, Heymsfield SB. Body composition and two-compartment model assumptions in male long distance runners. Med Sci Sports Exerc. 1994;26(3):392-7.

148. Perry AC, Shaw MH, Hsia L, Nash MS, Kaplan T, Signorile JF et al. Plasma lipid levels in active and sedentary premenopausal females. Int J Sports Med. 1992;13(3):210-5.

149. Petersen HL, Peterson CT, Reddy MB, Hanson KB, Swain JH, Sharp RL et al. Body composition, dietary intake, and iron status of female collegiate swimmers and divers. Int J Sport Nutr Exerc Metab. 2006;16(3):281-95.

150. Petibois C, Cassaigne A, Gin H, Deleris G. Lipid profile disorders induced by long-term cessation of physical activity in previously highly endurance-trained subjects. J Clin Endocrinol Metab. 2004;89(7):3377-84.

151. Pettersson U, Stalnacke B, Ahlenius G, Henriksson-Larsen K, Lorentzon R. Low bone mass density at multiple skeletal sites, including the appendicular skeleton in amenorrheic runners. Calcif Tissue Int. 1999;64(2):117-25.

152. Pettersson U, Alfredson H, Nordstrom P, Henriksson-Larsen K, Lorentzon R. Bone mass in female cross-country skiers: relationship between muscle strength and different BMD sites. Calcif Tissue Int. 2000;67(3):199-206.

153. Pfeiffer B, Stellingwerff T, Hodgson AB, Randell R, Pottgen K, Res P et al. Nutritional intake and gastrointestinal problems during competitive endurance events. Med Sci Sports Exerc. 2012;44(2):344-51.

154. Pichard C, Kyle UG, Gremion G, Gerbase M, Slosman DO. Body composition by x-ray absorptiometry and bioelectrical impedance in female runners. Med Sci Sports Exerc. 1997;29(11):1527-34.

155. Popovic M, Puchner S, Endler G, Foraschik C, Minar E, Bucek RA. The effects of endurance and recreational exercise on subclinical evidence of atherosclerosis in young adults. Am J Med Sci. 2010;339(4):332-6.

156. Poehlman ET, Gardner AW, Ades PA, Katzman-Rooks SM, Montgomery SM, Atlas OK et al. Resting energy metabolism and cardiovascular disease risk in resistance-trained and aerobically trained males. Metab. 1992;41(12):1351-60.

157. Poehlman ET, McAuliffe TL, Van Houten DR, Danforth E, Jr. Influence of age and endurance training on metabolic rate and hormones in healthy men. Am J Physiol. 1990;259(1 Pt 1):E66-72.

158. Procter-Gray E, Cobb KL, Crawford SL, Bachrach LK, Chirra A, Sowers M et al. Effect of oral contraceptives on weight and body composition in young female runners. Med Sci Sports Exerc. 2008;40(7):1205-12.

159. Proctor DN, Sinning WE, Bredle DL, Joyner MJ. Cardiovascular and peak VO2 responses to supine exercise: effects of age and training status. Med Sci Sports Exerc. 1996;28(7):892-9.

160. Quinn TJ, Manley MJ, Aziz J, Padham JL, MacKenzie AM. Aging and factors related to running economy. J Strength Cond Res. 2011;25(11):2971-9.

161. Quintas ME, Ortega RM, Lopez-Sobaler AM, Garrido G, Requejo AM. Influence of dietetic and anthropometric factors and of the type of sport practised on bone density in different groups of women. Eur J Clin Nutr. 2003;57 Suppl 1:S58-62.

162. Ratcliff L, Gropper SS, White BD, Shannon DM, Huggins KW. The influence of habitual exercise training and meal form on diet-induced thermogenesis in college-age men. Int J Sport Nutr Exerc Metab. 2011;21(1):11-8.

163. Rector RS, Rogers R, Ruebel M, Widzer MO, Hinton PS. Lean body mass and weight-bearing activity in the prediction of bone mineral density in physically active men. J Strength Cond Res. 2009;23(2):427-35.

164. Rejc E, Lazzer S, Antonutto G. Energy expenditure and dietary intake of athletes during an ultraendurance event developed by hiking, cycling and mountain climbing. J Sports Med Phys Fitness. 2010;50(3):296-302.

165. Rickenlund A, Carlstrom K, Ekblom B, Brismar TB, von Schoultz B, Hirschberg AL. Hyperandrogenicity is an alternative mechanism underlying oligomenorrhea or amenorrhea in female athletes and may improve physical performance. Fertil Steril. 2003;79(4):947-55.

166. Rico H, Revilla M, Villa LF, Gomez-Castresana F, Alvarez del Buergo M. Body composition in postpubertal boy cyclists. J Sports Med Phys Fitness. 1993;33(3):278-81.

167. Rico H, Revilla M, Hernandez ER, Gomez-Castresana F, Villa LF. Bone mineral content and body composition in postpubertal cyclist boys. Bone. 1993;14(2):93-5.

168. Riddell MC, Partington SL, Stupka N, Armstrong D, Rennie C, Tarnopolsky MA. Substrate utilization during exercise performed with and without glucose ingestion in female and male endurance trained athletes. Int J Sport Nutr Exerc Metab. 2003;13(4):407-21.

169. Riley-Hagan M, Peshock RM, Stray-Gundersen J, Katz J, Ryschon TW, Mitchell JH. Left ventricular dimensions and mass using magnetic resonance imaging in female endurance athletes. Am J Cardiol. 1992;69(12):1067-74.

170. Robertson JD, Maughan RJ, Duthie GG, Morrice PC. Increased blood antioxidant systems of runners in response to training load. Clin Sci (Lond). 1991;80(6):611-8.

171. Rogosheske V, Serfass RC, Kelly JM, Ainsworth B, Stull GA. Prediction of body fat in adult female runners. Clin Kinesiol. 1994;47(4):90-6.

172. Rokitzki L, Hinkel S, Klemp C, Cufi D, Keul J. Dietary, serum and urine ascorbic acid status in male athletes. Int J Sports Med. 1994;15(7):435-40.

173. Ross ML, Stephens B, Abbiss CR, Martin DT, Laursen PB, Burke LM. Fluid balance, carbohydrate ingestion, and body temperature during men's stage-race cycling in temperate environmental conditions. Int J Sports Physiol Perform. 2014;9(3):575-82.

174. Rossi FE, Ricci-Vitor AL, Sabino JPJ, Vanderlei LCM, Freitas Jr IF. Autonomic modulation and its relation with body composition in swimmers. J Strength Cond Res. 2014;28(7):2047-53.

175. Roy HJ, Lovejoy JC, Keenan MJ, Bray GA, Windhauser MM, Wilson JK. Substrate oxidation and energy expenditure in athletes and nonathletes consuming isoenergetic high- and low-fat diets. Am J Clin Nutr. 1998;67(3):405-11.

176. Rutherford OM. Spine and total body bone mineral density in amenorrheic endurance athletes. J Appl Physiol. 1993;74(6):2904-8.

177. Ryan AS, Nicklas BJ, Elahi D. A cross-sectional study on body composition and energy expenditure in women athletes during aging. Am J Physiol. 1996;271(5 Pt 1):E916-21.

178. Sanchez-Benito JL, Sanchez-Soriano E, Suarez JG. Unbalanced intake of fats and minerals associated with hypertension risk in young cyclists. Nutr Hosp. 2007;22(5):552-9.

179. Sanchez-Benito JL, Sanchez Soriano E. The excesive intake of macronutrients: does it influence the sports performances of young cyclists? Nutr Hosp. 2007;22(4):461-70.

180. Saunders MJ, Blevins JE, Broeder CE. Effects of hydration changes on bioelectrical impedance in endurance trained individuals. Med Sci Sports Exerc. 1998;30(6):885-92.

181. Schmidt WD, Hyner GC, Lyle RM, Corrigan D, Bottoms G, Melby CL. The effects of aerobic and anaerobic exercise conditioning on resting metabolic rate and the thermic effect of a meal. Int J Sport Nutr. 1994;4(4):335-46.

182. Schneider CM, Klich WJ, Insull W, Ferrington DA. Body composition and plasma lipoprotein profile of male and female swimming and track athletes. Sports Med Train Rehab. 1992;3(1):13-8.

183. Schtscherbyna A, Soares EA, de Oliveira FP, Ribeiro BG. Female athlete triad in elite swimmers of the city of Rio de Janeiro, Brazil. Nutrition. 2009;25(6):634-9.

184. Schulz LO, Nyomba BL, Alger S, Anderson TE, Ravussin E. Effect of endurance training on sedentary energy expenditure measured in a respiratory chamber. Am J Physiol. 1991;260(2 Pt 1):E257-61.

185. Sherk VD, Barry DW, Villalon KL, Hansen KC, Wolfe P, Kohrt WM. Bone loss over 1 year of training and competition in female cyclists. Clin J Sport Med. 2014;24(4):331-6.

186. Shimizu K, Suzuki N, Nakamura M, Aizawa K, Imai T, Suzuki S et al. Mucosal immune function comparison between amenorrheic and eumenorrheic distance runners. J Strength Cond Res. 2012;26(5):1402-6.

187. Shing CM, Webb JJ, Driller MW, Williams AD, Fell JW. Circulating adiponectin concentration and body composition are altered in response to high-intensity interval training. J Strength Cond Res. 2013;27(8):2213-8.

188. Singh A, Deuster PA, Day BA, Moser-Veillon PB. Dietary intakes and biochemical markers of selected minerals: comparison of highly trained runners and untrained women. J Am Coll Nutr. 1990;9(1):65-75.

189. Singh A, Evans P, Gallagher KL, Deuster PA. Dietary intakes and biochemical profiles of nutritional status of ultramarathoners. Med Sci Sports Exerc. 1993;25(3):328-34.

190. Sjodin AM, Forslund AH, Westerterp KR, Andersson AB, Forslund JM, Hambraeus LM. The influence of physical activity on BMR. Med Sci Sports Exerc. 1996;28(1):85-91.

191. Skarda ST, Burge MR. Prospective evaluation of risk factors for exercise-induced hypogonadism in male runners. West J Med. 1998;169(1):9-12.

192. Slater GJ, Rice AJ, Sharpe K, Tanner R, Jenkins D, Gore CJ et al. Impact of Acute Weight Loss and/or Thermal Stress on Rowing Ergometer Performance. Medicine & Science in Sports & Exercise. 2005;37(8):1387-94.

193. Slattery KM, Coutts AJ, Wallace LK. Nutritional practices of elite swimmers during an intensified training camp: with particular reference to antioxidants. J Sports Med Phys Fitness. 2012;52(5):501-5.

194. Smathers AM, Bemben MG, Bemben DA. Bone density comparisons in male competitive road cyclists and untrained controls. Med Sci Sports Exerc. 2009;41(2):290-6.

195. Smith R, Rutherford OM. Spine and total body bone mineral density and serum testosterone levels in male athletes. Eur J Appl Physiol Occup Physiol. 1993;67(4):330-4.

196. Snead DB, Stubbs CC, Weltman JY, Evans WS, Veldhuis JD, Rogol AD et al. Dietary patterns, eating behaviors, and bone mineral density in women runners. Am J Clin Nutr. 1992;56(4):705-11.

197. Snow CM, Rosen CJ, Robinson TL. Serum IGF-I is higher in gymnasts than runners and predicts bone and lean mass. Med Sci Sports Exerc. 2000;32(11):1902-7.

198. Soot T, Jurimae T, Jurimae J. Relationships between bone mineral density, insulin-like growth factor-1 and sex hormones in young females with different physical activity. J Sports Med Phys Fitness. 2006;46(2):293-7.

199. Soot T, Jurimae T, Jurimae J. Areal bone density in young females with different physical activity patterns: relationships with plasma leptin and body composition. J Sports Med Phys Fitness. 2007;47(1):65-9.

200. Soria M, Gonzalez-Haro C, Lopez-Colon JL, Llorente MT, Escanero JF. Submaximal exercise intensities do not provoke variations in plasma magnesium concentration in well-trained euhydrated endurance athletes with no magnesium deficiency. Magnes Res. 2011;24(2):36-44.

201. Soultanakis H, Platanou T. Effect of dietary carbohydrate content on blood glucose levels of swimmers during training. J Sports Med Phys Fitness. 2008;48(3):335-40.

202. Stewart AD, Hannan J. Total and regional bone density in male runners, cyclists, and controls. Med Sci Sports Exerc. 2000;32(8):1373-7.

203. Stöggl T, Hébert-Losier KIM, Holmbarg H-C. Do anthropometries, biomechanics, and laterality explain V1 side preference in skiers? Med Sci Sports Exerc. 2013;45(8):1569-76.

204. Stuempfle KJ, Lehmann DR, Case HS, Bailey S, Hughes SL, McKenzie J et al. Hyponatremia in a cold weather ultraendurance race. Alaska Med. 2002;44(3):51-5.

205. Stuempfle KJ, Lehmann DR, Case HS, Hughes SL, Evans D. Change in serum sodium concentration during a cold weather ultradistance race. Clin J Sport Med. 2003;13(3):171-5.

206. Stuempfle KJ, Hoffman MD, Hew-Butler T. Association of Gastrointestinal Distress in Ultramarathoners With Race Diet. International Journal of Sport Nutrition & Exercise Metabolism. 2013;23(2):103-9.

207. Stray-Gundersen J, Denke MA, Grundy SM. Influence of lifetime cross-country skiing on plasma lipids and lipoproteins. Med Sci Sports Exerc. 1991;23(6):695-702.

208. Strid H, Simren M, Storsrud S, Stotzer PO, Sadik R. Effect of heavy exercise on gastrointestinal transit in endurance athletes. Scand J Gastroenterol. 2011;46(6):673-7.

209. Sudi K, Jurimae J, Payerl D, Pihl E, Moller R, Tafeit E et al. Relationship between subcutaneous fatness and leptin in male athletes. Med Sci Sports Exerc. 2001;33(8):1324-9.

210. Suetta C, Kanstrup IL, Fogh-Andersen N. Haematological status in elite long-distance runners: influence of body composition. Clin Physiol. 1996;16(6):563-74.

211. Sutherland WH, Nye ER, Macfarlane DJ, Robertson MC, Williamson SA. Fecal bile acid concentration in distance runners. Int J Sports Med. 1991;12(6):533-6.

212. Sutherland WH, Nye ER, Macfarlane DJ, Williamson SA, Robertson MC. Cholesterol metabolism in distance runners. Clin Physiol. 1992;12(1):29-37.

213. Syrotuik DG, Game AB, Gillies EM, Bell GJ. Effects of Creatine Monohydrate Supplementation During Combined Strength and High Intensity Rowing Training on Performance. Canadian Journal of Applied Physiology. 2001;26(6):527-42.

214. Taaffe DR, Marcus R. Regional and total body bone mineral density in elite collegiate male swimmers. J Sports Med Phys Fitness. 1999;39(2):154-9.

215. Taaffe DR, Snow-Harter C, Connolly DA, Robinson TL, Brown MD, Marcus R. Differential effects of swimming versus weight-bearing activity on bone mineral status of eumenorrheic athletes. J Bone Miner Res. 1995;10(4):586-93.

216. Taipale RS, Mikkola J, Salo T, Hokka L, Vesterinen V, Kraemer WJ et al. Mixed maximal and explosive strength training in recreational endurance runners. J Strength Cond Res. 2014;28(3):689-99.

217. Tarnopolsky MA, Atkinson SA, Phillips SM, MacDougall JD. Carbohydrate loading and metabolism during exercise in men and women. J Appl Physiol (1985). 1995;78(4):1360-8.

218. Tarnopolsky MA, Bosman M, Macdonald JR, Vandeputte D, Martin J, Roy BD. Postexercise protein-carbohydrate and carbohydrate supplements increase muscle glycogen in men and women. J Appl Physiol (1985). 1997;83(6):1877-83.

219. Thompson J, Manore MM, Skinner JS. Resting metabolic rate and thermic effect of a meal in low- and adequate-energy intake male endurance athletes. Int J Sport Nutr. 1993;3(2):194-206.

220. Thompson JL, Manore MM, Skinner JS, Ravussin E, Spraul M. Daily energy expenditure in male endurance athletes with differing energy intakes. Medicine & Science in Sports & Exercise. 1995;27(3):347-54.

221. Thompson J, Manore MM. Predicted and measured resting metabolic rate of male and female endurance athletes. J Am Diet Assoc. 1996;96(1):30-4.

222. Tomten SE, Falch JA, Birkeland KI, Hemmersbach P, Hostmark AT. Bone mineral density and menstrual irregularities. A comparative study on cortical and trabecular bone structures in runners with alleged normal eating behavior. Int J Sports Med. 1998;19(2):92-7.

223. Tomten SE, Hostmark AT. Serum vitamin E concentration and osmotic fragility in female long-distance runners. J Sports Sci. 2009;27(1):69-76.

224. Tremblay A, Pinsard D, Coveney S, Catellier C, Laferriere G, Richard D et al. Counterregulatory response to insulin-induced hypoglycemia in trained and nontrained humans. Metab. 1990;39(11):1138-43.

225. Tuuri G, Loftin M, Oescher J. Association of swim distance and age with body composition in adult female swimmers. Med Sci Sports Exerc. 2002;34(12):2110-4.

226. Vanheest JL, Rodgers CD, Mahoney CE, De Souza MJ. Ovarian suppression impairs sport performance in junior elite female swimmers. Med Sci Sports Exerc. 2014;46(1):156-66.

227. Vogt S, Heinrich L, Schumacher YO, Grosshauser M, Blum A, Konig D et al. Energy intake and energy expenditure of elite cyclists during preseason training. Int J Sports Med. 2005;26(8):701-6.

228. Warren GL, Cureton KJ, Dengel DR, Graham RE, Ray CA. Is the gender difference in peak Vo2 greater for arm than leg exercise? Eur J Appl Physiol Occup Physiol. 1990;60(2):149-54.

229. Weight LM, Byrne MJ, Jacobs P. Haemolytic effects of exercise. Clin Sci (Lond). 1991;81(2):147-52.

230. Weight LM, Jacobs P, Noakes TD. Dietary iron deficiency and sports anaemia. Br J Nutr. 1992;68(1):253-60.

231. Weitkunat T, Knechtle B, Knechtle P, Rust CA, Rosemann T. Body composition and hydration status changes in male and female open-water swimmers during an ultra-endurance event. J Sports Sci. 2012;30(10):1003-13.

232. Wesche MF, Wiersinga WM. Relation between lean body mass and thyroid volume in competition rowers before and during intensive physical training. Horm Metab Res. 2001;33(7):423-7.

233. Wilber RL, Moffatt RJ, Scott BE, Lee DT, Cucuzzo NA. Influence of water run training on the maintenance of aerobic performance. Med Sci Sports Exerc. 1996;28(8):1056-62.

234. Williams C, Brewer J, Walker M. The effect of a high carbohydrate diet on running performance during a 30-km treadmill time trial. Eur J Appl Physiol Occup Physiol. 1992;65(1):18-24.

235. Williford HN, Olson MS, Keith RE, Barksdale JM, Blessing DL, Wang NZ et al. Iron status in women aerobic dance instructors. Int J Sport Nutr. 1993;3(4):387-97.

236. Wilmore JH, Wambsgans KC, Brenner M, Broeder CE, Paijmans I, Volpe JA et al. Is there energy conservation in amenorrheic compared with eumenorrheic distance runners? J Appl Physiol. 1992;72(1):15-22.

237. Withers RT, Smith DA, Chatterton BE, Schultz CG, Gaffney RD. A comparison of four methods of estimating the body composition of male endurance athletes. Eur J Clin Nutr. 1992;46(11):773-84.

238. Withers RT, van Der Ploeg G, Finn JP. Oxygen deficits incurred during 45, 60, 75 and 90-s maximal cycling on an air-braked ergometer. Eur J Appl Physiol Occup Physiol. 1993;67(2):185-91.

239. Wulff Helge E, Melin A, Waaddegaard M, Kanstrup IL. BMD in elite female triathletes is related to isokinetic peak torque without any association to sex hormone concentrations. J Sports Med Phys Fitness. 2012;52(5):489-500.

240. Zaccaria M, Ermolao A, Brugin E, Bergamin M. Plasma leptin and energy expenditure during prolonged, moderate intensity, treadmill exercise. J Endocrinol Invest. 2013;36(6):396-401.

241. Zietz B, Schnabl S, Nerlich M, Schoelmerich J, Schaeffler A. Nutritional composition in different training stages in young female athletes (swimming) and association with leptin, IGF-1 and estradiol. Exp Clin Endocrinol Diabetes. 2009;117(6):283-8.
